# The Roles of *Arabidopsis* CDF2 in Transcriptional and Posttranscriptional Regulation of Primary MicroRNAs

**DOI:** 10.1371/journal.pgen.1005598

**Published:** 2015-10-16

**Authors:** Zhenfei Sun, Tongtong Guo, Yin Liu, Qi Liu, Yuda Fang

**Affiliations:** National Key Laboratory of Plant Molecular Genetics, Shanghai Institute of Plant Physiology and Ecology, Shanghai Institutes for Biological Sciences, Chinese Academy of Sciences, Shanghai, China; University of California Riverside, UNITED STATES

## Abstract

The precise regulation of microRNA (miRNA) transcription and processing is important for eukaryotic development. Plant miRNAs are first transcribed as stem-loop primary miRNAs (pri-miRNAs) by RNA polymerase II,then cleaved in the nucleus into mature miRNAs by Dicer-like 1 (DCL1). We identified a cycling DOF transcription factor, CDF2, which interacts with DCL1 and regulates the accumulation of a population of miRNAs. CDF2 binds directly to the promoters of some miRNAs and works as a transcription activator or repressor for these miRNA genes. CDF2 binds preferentially to the pri-miRNAs regulated by itself and affects DCL1-mediated processing of these pri-miRNAs. Genetically, CDF2 works in the same pathway as miR156 or miR172 to control flowering. We conclude that CDF2 regulates a group of pri-miRNAs at both the transcriptional and posttranscriptional levels to maintain proper levels of their mature miRNAs to control plant development.

## Author Summary

CDFs were identified to play roles in the blue light signaling. This study reveals that CDF2 acts as a transcriptional activator or repressor of a group of microRNA (miRNA) genes and binds to the pri-miRNA transcripts. This study demonstrates that CDF2 interacts with the Dicer-like 1 (DCL1) complex and suppresses the processing of primary miRNAs. Genetic analysis shows that CDF2 works in the same pathway as miR156 or miR172 to control flowering. The finding that the miRNA accumulation is regulated by a factor at both the transcriptional and posttranscriptional levels may have a broad impact on the miRNA biogenesis field. The regulation of miRNA abundance by CDF2 sheds light on the roles of miRNAs in the light signaling pathways.

## Introduction

The 20–22 nt-long microRNAs (miRNAs) are essential regulators in many biological processes in almost all eukaryotes [1]. miRNAs are processed from stem-loop primary transcripts (pri-miRNAs), which are transcribed by DNA-dependent RNA polymerase II [2]. In plants, pri-miRNAs are captured by the RNA-binding protein dawdle (DDL), which presumably stabilizes pri-miRNAs [3], then processed in a two-step manner in the nucleus into mature miRNAs by a single RNAse III enzyme, dicer-like1 (DCL1) [4] and its partner, the double-strand RNA-binding domain (dsRBD) protein hyponastic leaves1 (HYL1) [5]. Other proteins involved include the zinc finger domain protein serrate (SE) [6], C-terminal domain phosphatase-like 1 (CPL1) [7], the nuclear cap-binding complex [8], TOUGH [9], and MOS2 [10].

Cycling DOF transcription factors (CDFs) are members of the DNA-binding with one finger (Dof) gene family [11,12]. The roles of several Dof-type zinc finger transcription factors are known. The *Dof affecting germination 1* (*dag1*) mutant does not require light for germination [13], in contrast to the *dag2* mutant [14]. Overexpression of AtOBP3 in *Arabidopsis* resulted in altered root development and yellowish leaves [15]. AtCOG1 is a negative regulator in the phyA and phyB signaling pathways [16]. CDFs are involved in photoperiodic flowering; each *cdf* mutant exhibits an early flowering phenotype [11]. Dofs may also function in flowering regulation in bamboo [17]. The blue light photoreceptor FKF1 interacts with CDF transcription factors for poly-ubiquitination-dependent degradation [18].

Several factors were recently identified to be involved in miRNA transcription and processing. NOT2 coordinates *MIR* transcription and efficient DCL1 recruitment in *Arabidopsis* miRNA biogenesis [19]. The transcription factor CDC5 reduces *MIR* promoter activity, interacts with the DCL1 complex, and is required for miRNA processing in *Arabidopsis* [20]. MeCP2 inhibits gene transcription and also suppresses miRNA processing by binding to the RNA-binding domains of human DGCR8 [21]. Drosha and DCL4 modulate transcription termination of HIV-1 and FCA, respectively [22,23]. Therefore, it is of interest to address whether and how the transcription and processing of pri-miRNAs are coordinated.

In this study, we identified the transcription factor CDF2 that interacts with DCL1. We uncovered the roles of CDF2 in miRNA biogenesis at both the transcription and post-transcription levels to regulate plant flowering.

## Results and Discussion

### CDF2 interacts with DCL1

**Fig 1:**
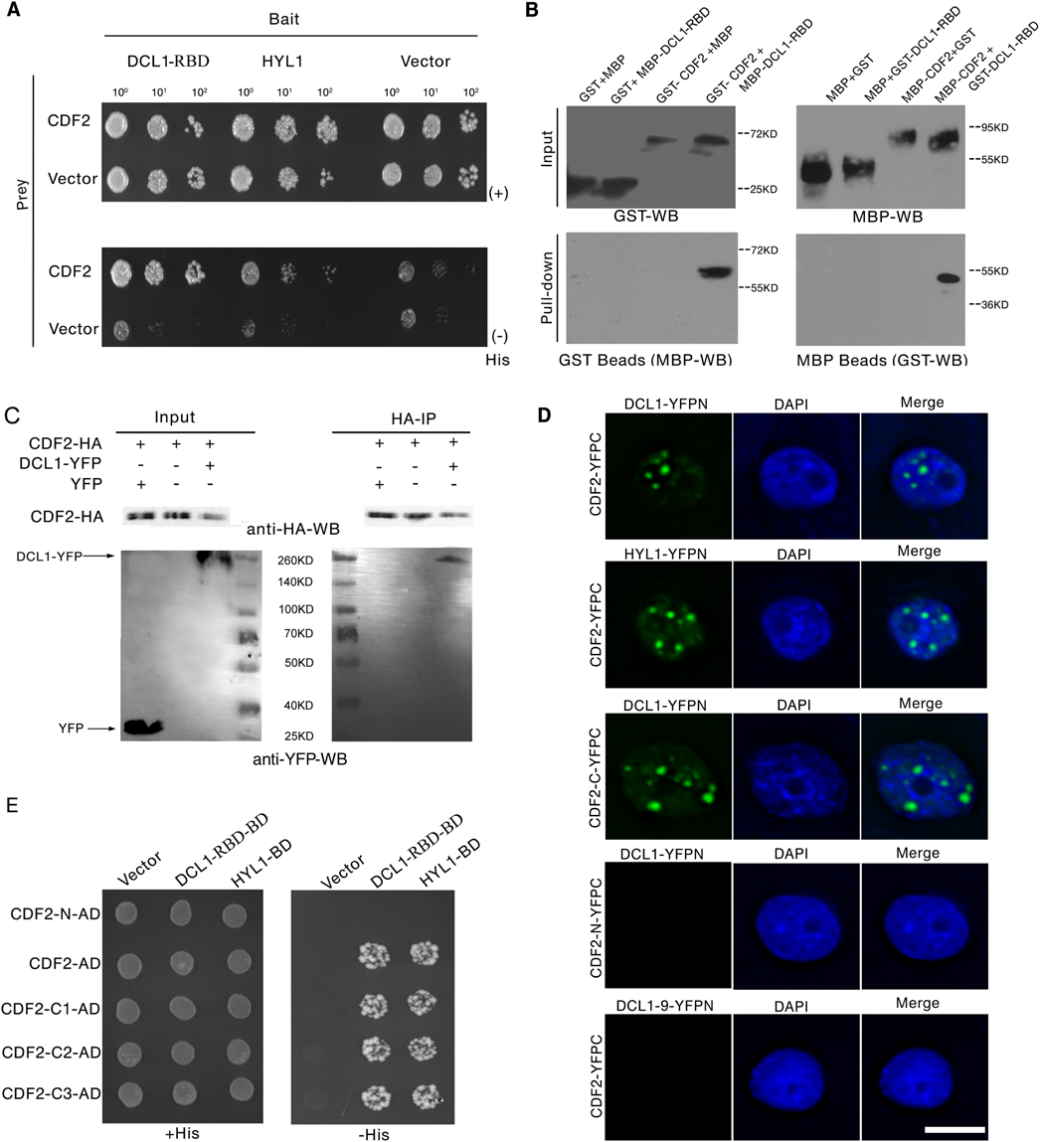
CDF2 interacts with DCL1. (**A**) Yeast two hybrid assays show the interactions between CDF2 and DCL1-RBD or HYL1. Co-transformed yeast colonies were spotted on the selective SC medium minus Trp and Leu, and then grown on SC medium minus His, Trp, and Leu supplemented with 5mM 3-amino-1, 2, 4-triazole (3-AT). (**B**) GST and MBP pull-down assays show the interaction between CDF2 and DCL1. (**C**) Co-IP assay shows the interaction between CDF2 and DCL1. The protein extracts from 22-day-old *Arabidopsis* plants coexpressing *pCDF2*::*CDF2-HA* and *pDCL1*::*DCL1-YFP* were incubated with anti-HA–conjugated agarose. The pellet was analyzed by immunoblotting with anti-HA and anti-GFP antibodies. (**D**) Bimolecular Fluorescence Complementation (BiFC) assays show that CDF2 or the C-terminal fragment of CDF2 (CDF2-C) interact with DCL1/HYL1 in D-bodies, while no interactions were observed between CDF2 and DCL1-9 or the N-terminal fragment of CDF2 (CDF2-N) and DCL1. Scale bar = 10μm. (**E**) Yeast two hybrid assays show that the C terminal fragment of CDF2 interacts with DCL1-RBD. C1, aa 189–457; C2, aa 271–457; C3, aa 360–457.

To identify new components involved in regulation of miRNA biogenesis, we performed a yeast two-hybrid screening for proteins that interact with the two C-terminal dsRBDs of DCL1 (DCL1-RBD, aa 1733–1910), which complement the phenotypes of *hyl1* mutant and are important for protein–protein interactions and pri-miRNA bindings [24, 25]. Among the obtained 54 independent prey clones, 15 represented DCL1, which is consistent with the findings of previous studies that DCL1 can interact with itself [24,25]. Four independent prey clones contained various C-terminal fragments of CDF2. We then examined the interactions between full length of DCL1/HYL1 and CDF2 by yeast two-hybrid assays, the results showed that CDF2 can interact with DCL1 and HYL1 (Fig 1A).

We confirmed the interaction between DCL1 and CDF2 using maltose-binding protein (MBP) and glutathione S-transferase (GST) pull-down assays. The purified fusion proteins MBP-CDF2, MBP-DCL1-RBD, GST-CDF2, and GST-DCL1-RBD were clearly observed on a sodium dodecyl sulfate polyacrylamide gel electrophoresis (SDS-PAGE) gel stained with coomassie dye (S1 Fig) The GST and MBP proteins were included in parallel assays as negative controls (Fig 1B). The pull-down results showed that the fusion proteins MBP-DCL1-RBD and GST-DCL1-RBD interact with GST-CDF2 and MBP-CDF2, respectively, whereas no interactions with the controls were observed (Fig 1B).

Co-IP experiments were then performed to confirm the interaction *in vivo*. Total proteins were extracted from 22-day-old *Arabidopsis* plants coexpressing hemagglutinin (HA)-labeled CDF2 (*pCDF2*::*CDF2-HA)* and yellow fluorescent protein (YFP)-labeled DCL1 (*pDCL1*::*DCL1-YFP*), which were generated by crossing *pCDF2*::*CDF2-HA* with *pDCL1*::*DCL1-YFP* transgenic lines [26] (S2A–S2D and S5B Figs). For a negative control, the proteins were extracted from the plants co-expressing *pCDF2*::*CDF2-HA/cdf2* and *p35S*::*YFP*. These proteins were then incubated with HA-conjugated agarose beads to immunoprecipitate DCL1. DCL1-containing complexes were separated by a sodium dodecyl sulfate polyacrylamide gel electrophoresis (SDS-PAGE) and immunoblotted with anti-green fluorescent protein (GFP) and anti-HA antibodies, respectively. CDF2 was observed to interact with DCL1 (Fig 1C).

Bimolecular Fluorescence Complementation (BiFC) assays were performed to further investigate the interaction between CDF2 and components in microprocessor. As shown in Fig 1D, we found that CDF2 interacts with DCL1/HYL1 in nuclear dicing bodies, possibly through the C-terminal fragment of CDF2 (CDF2-C). In contrast, no interactions were observed between CDF2 and DCL1-9, a C-terminal DsRBDs-truncated form of DCL1, or the N-terminal fragment of CDF2 (CDF2-N) and DCL1.

We then determined the fragment of CDF2 which interacts with DCL1 using a yeast two-hybrid assay. CDF2 contains an N-terminal Dof domain and a C-terminal fragment of unknown function. The results showed that the C-terminal fragments of CDF2 interacted with DCL1 as strongly as the full-length CDF2 (Fig 1D), We then narrowed down the fragment that interacts with DCL1, we found that the fragments of CDF2 (aa 361–398, aa 396–457, aa 396–421 and 385–400) failed to bind to DCL1 (S3 Fig), indicating that the interaction between CDF2 and DCL1 was mediated by the C-terminal region (aa 360–436) of CDF2.

### The accumulation of a group of miRNAs is affected by CDF2

**Fig 2:**
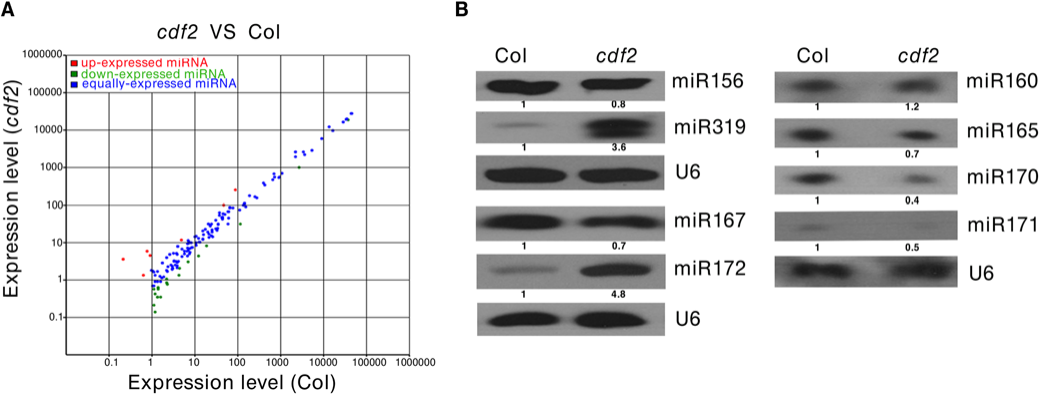
CDF2 is required for the accumulation of a group of miRNAs. (**A**) Comparision of miRNAs that are differently expressed in 22-day-old plant of Col and *cdf2* mutant. (**B**) Northern blots show the levels of miRNAs in seedlings of Col and *cdf2* mutant. U6 serves as a loading control.

As CDF2 interacts with the microprocessor DCL1/HYL1, we then investigated if miRNAs are regulated by CDF2. To this end, we applied high-throughput sequencing to analyze the global miRNAs in 22-day-old plants of Col and *cdf2*, using *dcl1-9* mutant as a control. Totally, 114,892,98, 123,492,11 and 130,812,02 reads were obtained from WT, *cdf2* and *dcl1-9*, and 13,233,29 (54.98%), 24,975,74 (75.97%) and 11,882,21 (53.42%) reads, representing 7,275,87, 18,973,61 and 6,346,98 distinct sequences, respectively, matched the *Arabidopsis* genome. The sequencing data for all known miRNAs were subjected to hierarchical clustering in an unsupervised manner to analyze the extent of differential miRNAs [27] (Figs 2A and S4, S1 and S2 Tables). At least 1.5-fold changes in the levels were observed for 72 of 195 miRNAs detected in both Col and the *cdf2* mutant. Among these, 52 (72%) were significantly downregulated, whereas 20 (28%) were upregulated. The small RNA-seq results were validated by northern blotting of miR156, miR319, miR167, miR172, miR160, miR165, miR170, and miR171 repressed or activated by CDF2, respectively (Figs 2B and S5A). In contrast, almost all of the miRNAs are downregulated in *dcl1-9* mutant (S4 Fig, S2 Table). To minimize the potential effect of different developmental stages between Col and *cdf2* mutant on the interpretation of our data, we performed northern blots using inflorescences of Col, *cdf2* and *pCDF2*::*CDF2-HA*/*cdf2*, which was generated by crossing of *cdf2* mutant with the *pCDF2*::*CDF2-HA*/Col line. We found that the expressional levels of these miRNAs are similar to those in 22-day-old seedlings. In addition, the *pCDF2*::*CDF2-HA*/*cdf2* line restored the phenotypes and miRNAs levels of *cdf2* mutant (S2B and S5B Figs), Taking together, we concluded that CDF2 regulates biogenesis of a population of miRNAs.

### CDF2 regulates the transcription of a population of primary miRNAs

**Fig 3:**
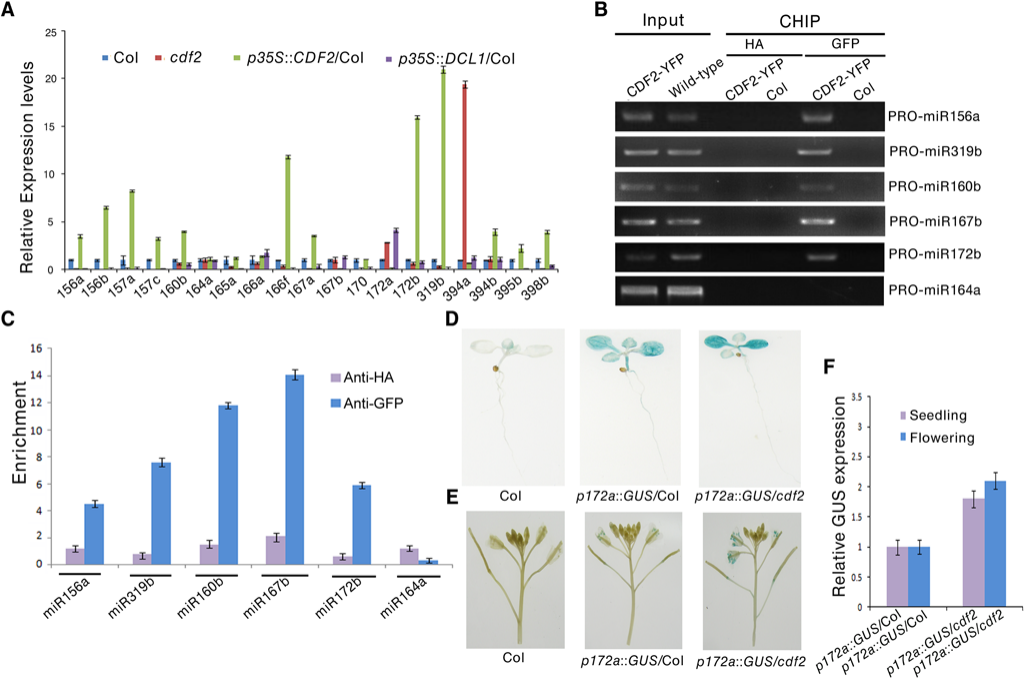
CDF2 acts as a transcription factor for some miRNA genes. (**A**) The relative levels of pri-miRNAs in Col, *cdf2*, *p35S*::*CDF2*/Col, *p35S*::*DCL1*/Col lines examined by real-time PCR. The relative fold changes were normalized to *ACTIN*. Data are given as means ± SD (n = 3). (**B**) ChIP-PCR analysis of five promoter fragments of miRNA genes in wild-type and *pCDF2*:*CDF2-YFP*/Col seedlings. ChIP assays were performed using the 22-day-old Col-0 and *pCDF2*::*CDF2-YFP*/Col seedlings expressing the CDF2-YFP fusion protein. DNA was amplified using primers specific to 6 miRNA promoter regions. (**C**) CHIP followed by real time PCR of 6 promoter fragments of miRNA genes in Col and *pCDF2*::*CDF2-YFP*/Col seedlings. Relative enrichment of fragments was calculated with HA antibodies as the control. Data are given as means ± SD (n = 3). (**D**) and (**E**) *pMIR172a*::*GUS* in Col, *cdf2* and *p35S*::*DCL1*/Col in seedlings (**D**) and flowers (**E**), respectively. Thirty plants containing GUS were analyzed for each of genotypes. (**F**) The transcript levels of GUS driven by *miR172b* promoter in Col, *cdf2* and *p35S*::*DCL1*/Col. GUS transcript levels were determined by qRT-PCR. Data are given as means ± SD (n = 3).

To address the molecular mechanism of the effect of CDF2 on miRNA abundance, we first examined the expressional levels of *DCL1* and *HYL1*, the two main components involved in miRNA biogenesis, in *cdf2* mutant and CDF2 overexpression lines (S6 Fig), the result shows that the expression levels of the genes are similar to those in Col. We then analyzed the levels of 19 pri-miRNAs, for which their mature miRNAs show at least 1.5-fold differences (S1 Table), by quantitative real-time PCR (qRT-PCR) in seedlings of *cdf2* mutant and CDF2-overexpressing (*p35S*::*CDF2*/Col) lines. As shown in Fig 3A, the relative levels of pri-miRNAs between *cdf2* and *p35S*::*CDF2*/Col are predominantly opposite for all 19 pri-miRNAs. Some of pri-miRNAs (pri-mi172a and pri-miR394a) are up-regulated, while others down-regulated in *cdf2* mutant (Fig 3A), indicating that CDF2 acts as both a transcriptional activator and repressor, which is similar to other Dof proteins, e.g., maize Dof2 exhibits either activation or repression activities on different promoters [28]; the barley Dof factor (prolamin-box binding factor) activates the transcription of B-hordein, but represses the transcription of cathepsin B-like protease [29]. The distinct roles of CDF2 in miRNA transcription may be defined by the components forming different transcriptional machinery to be recruited to different promoters.

To determine whether CDF2 binds to the promoters of these miRNA genes or not, we performed a chromatin immunoprecipitation-PCR (ChIP-PCR) assay using GFP antibody-precipitated chromatin from *pCDF2*::*CDF2-YFP* transgenic plant (S2A Fig). We focused on the promoters of miRNA genes for which the levels of pri-miRNAs changed in the *cdf2* mutant (Fig 3A, S2 Table). The promoter fragments of miRNA genes (including miR156a, miR319b, miR160b, miR167b, and miR172b) were amplified from GFP antibody-immunoprecipitated, but not from HA antibody-immunoprecipitated *pCDF2*::*CDF2-YFP*/Col samples (Figs 3B, 3C and S2A). Importantly, the promoter fragment of miR164a, which is not regulated by CDF2 (Fig 3A), was not amplified from GFP antibody-immunoprecipitated *pCDF2*::*CDF2-YFP*/Col (Fig 3B and 3C). In contrast, no apparent enrichment of fragments in Col was observed (Fig 3B).

To further test the effect of *cdf2* on the miRNA expression, we used a β-glucuronidase (*GUS*) reporter gene driven by the promoter of *miR172a* whose expression is repressed by CDF2 (Fig 3A). This system was previously used to determine the function of DDL, CDC5, and NOT2 in the regulation of miR gene transcription [3,19,20]. We crossed *cdf2* with transgenic plants containing *pmiR172a*::GUS. In the second (F2) generation, we obtained CDF2/CDF2, CDF2/*cdf2* and *cdf2/cdf2* genotypes containing *pMIR172a*::*GUS*. *GUS* was markedly increased in *cdf2/cdf2* compared to that in CDF2+ plants (Fig 3D and 3E). Quantitative RT-PCR analysis indicated that the *GUS* mRNA levels in *cdf2* mutant were increased at different developmental stages (Fig 3F). Together, we conclude that CDF2 regulates the transcription of a group of miRNA genes.

### CDF2 affects the post-transcriptional processing of a subset of primary miRNAs

**Fig 4:**
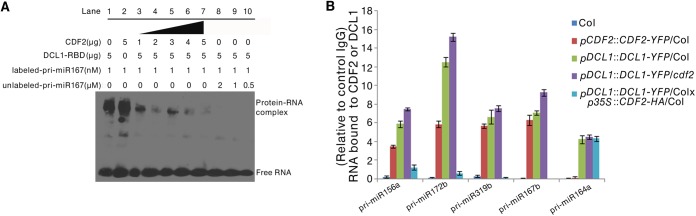
CDF2 binds to pri-miRNAs and affects the binding of DCL1 to pri-miRNAs. (**A**) RNA EMSAs show the effect of CDF2 on the binding of DCL1 to pri-miRNA167. (**B**) An RIP assay was performed using 22-day-old plants, RNA fragments were immunoprecipitated with the GFP antibody and subsequently subjected to qRT-PCR analysisfor the indicated pri-miRNAs. Data are given as means ± SD (n = 3).

To investigate the biological role of the interaction between CDF2 and DCL1 in miRNA biogenesis, we performed RNA competitive electrophoretic mobility shift assays to test whether CDF2 affects the well-known binding activity of DCL1-RBD to pri-miRNAs. The reactions were performed using a fixed DCL1-RBD concentration and increasing amounts of CDF2. Interestingly, similar to DCL1-RBD (Figs 4A, lane1, and S7A–S7D, lane1), CDF2 was also observed to bind to pri-miR167b (Figs 4A, lane2, and S7A-S7D, lane2), and the binding ability of DCL1-RBD to the pri-miRNA decreased as the concentration of CDF2 increased (Figs 4A and S7A–S7D, lane 3–7), indicating that the interaction between CDF2 and DCL1-RBD affects the binding of DCL1-RBD to the pri-miRNA *in vitro*. In addition, we also found that the binding of CDF2 to pri-miRNAs was mainly mediated by its C-terminal fragment (S7E Fig). To test the pri-miRNA binding of CDF2 *in vivo*, an RNA immunoprecipitation assay was performed. Using a GFP antibody, we immunoprecipitated CDF2-YFP and DCL1-YFP complexes from the 22-day-old seedlings of pCDF2::CDF2-YFP/Col (S2A Fig), pDCL1::DCL1-YFP/Col, pDCL1::DCL1-YFP/*cdf2*, and pDCL1::DCL1-YFP/Col X p35S::CDF2-HA/Col line which was generated by crossing pDCL1::DCL1-YFP with p35S::CDF2-HA transgenic lines, using Col as a negative control of plants. In parallel, an IgG antibody- immunoprecipitated samples from these lines were used as a negative control of the antibody. Interestingly, the immunoprecipitated CDF2-YFP complex (S8 Fig) contains pri-miR156a, pri-miR172b, pri-miR319b, and pri-miR167b, which have altered levels in the *cdf2* mutant, but not pri-miR164a, which showed no change in *cdf2* mutant (Fig 4B, S1 Table). In contrast, DCL1 was able to bind to all 5 pri-miRNAs examined (Fig 4B). The bindings of the DCL1 to pri-miRNAs increase in the pDCL1::DCL1-YFP/*cdf2* and decrease in the line of *pDCL1*::*DCL1-YFP*/Col X *p35S*::*CDF2-HA*/Col. These results suggested that CDF2 binds mainly to the CDF2-regulated pri-miRNAs *in vivo*, possibly due to that CDF2 has more accessibility to these pri-miRNAs transcribed with the aid of the transcription complex containing CDF2, this was supported by a recent report that transcription and processing of primary microRNAs are coupled by Elongator complex [30], resulting in that CDF2 only affects the processing of a subset of pri-miRNAs.

We also compared the the levels of steady-state pri-miRNAs among Col, *hyl1*, *cdf2* and *hyl1/cdf2* mutants (S9 Fig). Interestingly, for pri-miR164 which was not affected by CDF2 at both transcriptional and post-transcriptional levels, the amount of pri-miR164 in *hyl1* is similar to that in *hyl1/cdf2*. Other pri-miRNAs were regulated by HYL1 and CDF2 in different degrees (S9 Fig).

**Fig 5:**
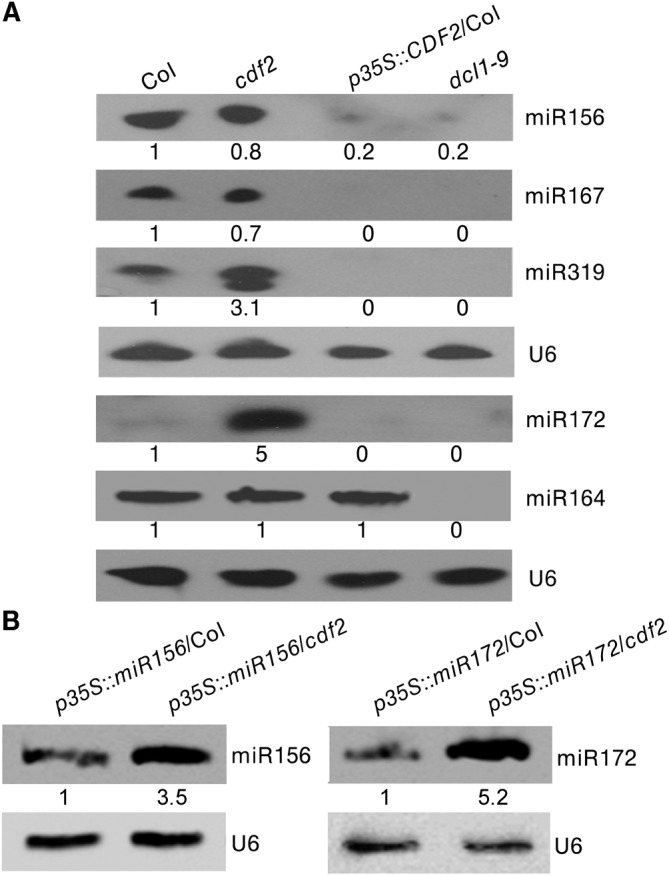
CDF2 suppresses the posttranscriptional processing of pri-miRNAs. (**A**) Northern blots show that overexpression of CDF2 reduce the accumulation of miRNAs indicated. (**B**) Northern blots show that the levels of miR156 and miR172 in *p35S*::miR156/Col and *p35S*::miR156/*cdf2* or *p35S*::miR172/Col and *p35S*::miR172/*cdf2* plants.

As CDF2 interacts with DCL1, and affects the binding of DCL1 to pri-miRNAs (Figs 4A and S7A–S7D), we then evaluated the role of CDF2 in DCL1-mediated miRNA processing *in vivo*. To this end, we compared the accumulation levels of five miRNAs, including miR156, miR167, miR319, and miR172 which are regulated by CDF2, and miR164 which is not regulated by CDF2, in Col, *cdf2*, *p35S*::*CDF2-YFP*/Col (S10A Fig), and *dcl1-9* lines. We found that the accumulation levels of miR156, miR167, miR319, and miR172 in *p35S*::*CDF2-YFP*/Col were significantly lower than that in *cdf2* mutant and Col lines (Fig 5A). In contrast, miR164 accumulation level in the *p35S*::*CDF2-YFP*/Col line is similar to that in Col, possibly due to that CDF2 does not bind to miRNA164 *in vivo* which is not regulated at the transcriptional level by CDF2 (Fig 4B). We also observed that the levels of *CDF2* and miRNAs in plants expressing CDF2-YFP under the control of its endogenous promoter (*pCDF2*::*CDF2-YFP*/Col) were very similar to those in Col (S10A and S10B Fig).

As CDF2 can affect the accumulation of miRNAs at both the transcriptional and post-transcriptional levels, to further test if CDF2 affects the post-transcriptional processing of pri-miRNAs or not, we crossed the transgenic lines of *p35S*::*miR156*/Col and *p35S*::*miR172*/Col with the *cdf2* mutant, then compared the accumulation levels of miRNA156 in *p35S*::*miR156*/Col and *p35S*::*miR156*/*cdf2* plants by northern blot (the miR156 transcripts were similar to each other in these two lines as shown in S11 Fig), and found that the accumulation level of miR156 is lower in *p35S*::*miR156*/Col than that in *p35S*::*miR156*/*cdf2* (Fig 5B). We also compared the levels of miRNA172 in *p35S*::*miR172*/Col and *p35S*::*miR172*/*cdf2* (the miR172 transcripts were similar to each other in these two lines as shown in S11 Fig), the results showed that the miR172 level is lower in *p35S*::*miR172*/Col than that in *p35S*::*miR172*/*cdf2* (Fig 5B), suggesting that CDF2 suppresses the post-transcriptional processing of miRNAs, possibly because CDF2 binds to these pri-miRNAs regulated by itself at transcriptional level (Fig 4B), and CDF2 interacts with DCL1/HYL1 which might sequester DCL1/HYL1, resulting in decreased miRNA processing by the DCL1/HYL1 microprocessor. In animals, several factors were found to inhibit the activities of Drosha and Dicer, including ADAR (adenosine deaminase acting on RNA) enzymes that participate in adenosine-to-inosine (A-to-I) RNA editing to prevent effective processing of specific pri-miRNAs by Drosha [31]; estrogen-bound estrogen receptor alpha, which associates with the Drosha complex and prevents the conversion of pri-miRNAs to pre-miRNAs [32]; LIN28, which blocks the accumulation of mature miRNAs by repression of both Drosha and Dicer activities [33,34,35]; and TUT4, which is recruited by Lin28 to specific pri-miRNAs, leading to 3′ terminal uridylation and degradation of pri-miRNAs [36,37]. MeCP2 suppresses miRNA processing by binding to the RNA-binding domains of human DGCR8 [21].

We then investigated the effect of CDF2 on the expressions of components involved in miRNA processing. As shown in S12 Fig, compared to those in wild type, the expressions of *SE*, *MOS2*, *CDC5*, *TOUGH*, *DDL*, *NOT2* and *CPL1* are down-regulated in cdf2 mutant and upregulated in the *CDF2* overexpression line, while the effect of CDF2 on the expression of *HYL1* and *DCL1* is not obviously. The result implicated that CDF2 might regulate biogenesis of miRNAs indirectly by regulating the expressions of miRNA processing proteins in addition to its direct roles in transcriptional and post-transcription regulations of pri-miRNAs. Similarly, Cho et al. [39] found that the immensely accumulated DCL1 level in *hyl1-2* and *se-1* mutants, and the increased level of SE in *hyl1-2* mutant. It is of interest to investigate the potential feedback regulatory network among components involved in miRNA biogenesis.

### CDF2 plays a role in flowering through a pathway same as miR156/miR172

**Fig 6:**
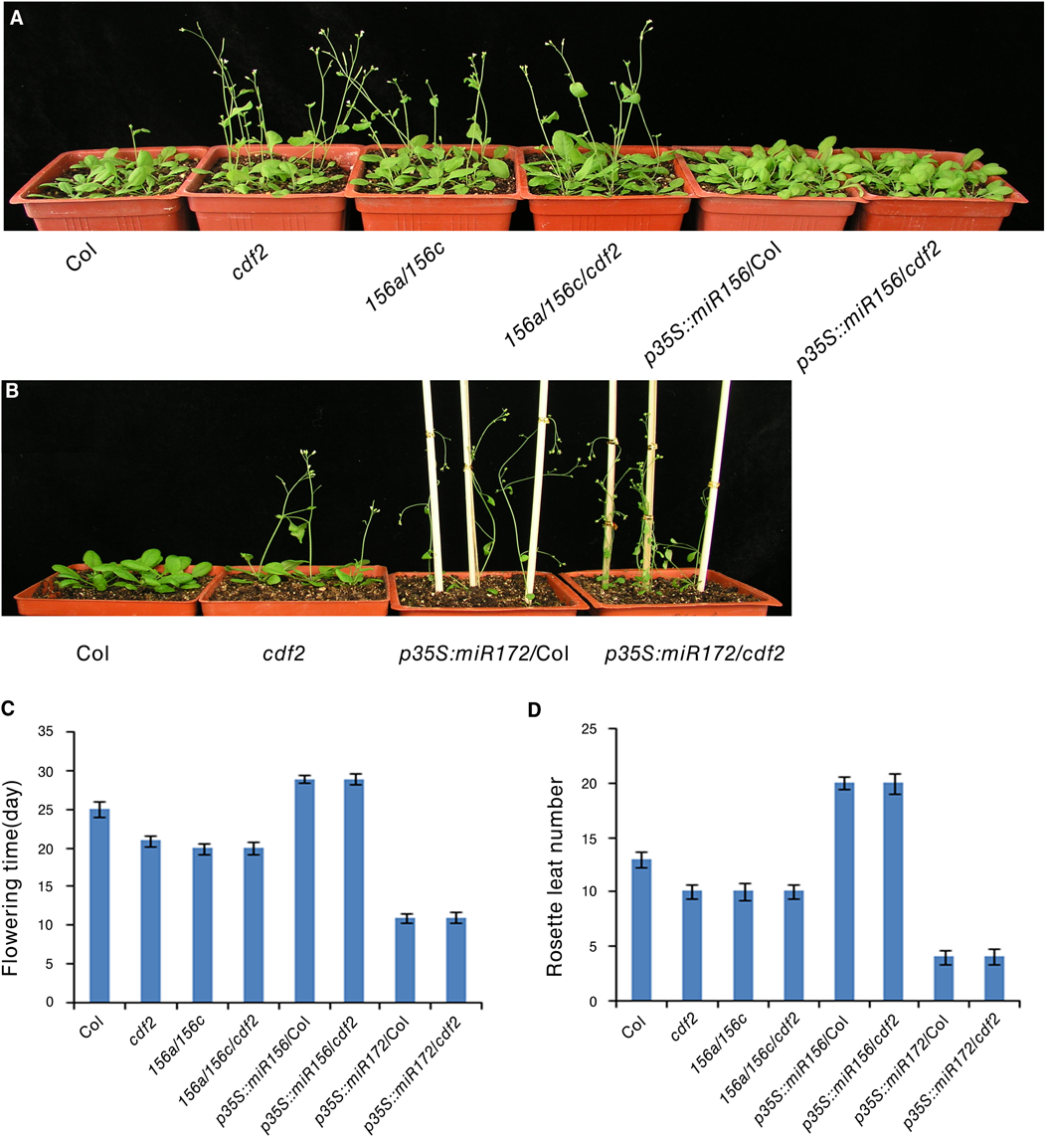
CDF2 is in the same pathway as miR156 and miR172 to regulate flowering. (**A**) and (**B**) 24-day-old plants of indicated genotypes grown in longday photoperiods (16 hours light, 8 hours dark). (**C**) The time of flowering at the time of flowering of the indicated genotypes shown in A and B. Data are mean ± SEM of 50 plants. (**D**) The number of rosette leaves at the time of flowering of the indicated genotypes shown in A and B. Data are means ± SEM of 50 plants.

Several CDF2-regulated miRNAs are involved in flowering, including miR156, miR160, miR167, miR172, miR165/miR166, and miR319 [38], we investigated if miRNAs contribute to the earlier flowering phenotype of *cdf2* mutant [11]. We focused on miR156 and miR172, two widely studied miRNAs [40] (S1 Table). It was reported that *miR156* mutant line flowers earlier and *miR172* mutant flowers later [38,41], in accordance to the early flowering phenotype of the *cdf2* mutant in which the level of miR156 decreases (S1 Table) and its target squamosa promoter binding protein-like (SPL) proteins increase (S13A Fig), whereas miR172 increases (S1 Table) and its target AP2 decreases in *cdf2* (S13B Fig) [42,43], supporting a role of CDF2 in miRNA regulation. We then first investigated the genetic relationship between CDF2 and miR156. To this end, we generated *miR156a/miR156c/cdf2* triple mutant and found that the flowering time of this triple mutant flowered not earlier than either *cdf2* or *miR156a/ miR156c* lines (Fig 6A, 6C and 6D), suggesting that *CDF2* and *miR156* regulate flowering through the same pathway. In addition, miR156 overexpression in *cdf2* mutant showed the same late flowering phenotype as miR156 overexpression in Col (Figs 6A, 6C, 6D, S10 and S13A), indicating that miR156 acts genetically downstream of CDF2 to regulate flowering. Next, we overexpressed miR172 in *cdf2* mutant and found that this overexpression line (*p35S*::*miR172*/*cdf2*) did not display earlier flowering phenotype than *cdf2* mutant or miR172 overexpressed line in Col (Figs 6B–6D, S10 and S13B), suggesting that *cdf2* and *miR172* regulate flowering through the same signaling pathway. Together, these results indicated the role of CDF2 in miR156 activation and miR172 suppression which participate in the flowering time control. We next generated *cdf2/hyl1* double mutants, we found that the flowering of *cdf2/hyl1-2* plants was similar to the *hyl1*-2 single mutant [11,44] (S14A–S14C Fig), suggesting that *CDF2* and *HYL1* regulate flowering through the same pathway. CDF2 was reported to be a transcriptional repressor of *constans* (*CO*) to control flowering, our results thus revealed a new pathway for regulating the flowering through CDF2 and miRNAs which might be independent of CO.

In summary, we have revealed the previously unknown roles of CDF2 in the transcriptional and post-transcriptional regulation of a subset of pri-miRNAs. At the transcriptional level, CDF2 works as a transcription activator or repressor to regulate the transcription of a group of miRNA genes. We also found that CDF2 interacts with DCL1 and preferentially binds to the pri-miRNAs regulated transcriptionally by itself *in vivo*, which might affect the binding of DCL1 to pri-miRNAs or sequester the microprocessor DCL1/HYL1, resulting in the influence on pri-miRNA processing. By transcriptional and post-transcriptional regulation of a group pri-miRNAs, CDF2 may precisely regulate a population of miRNAs to maintain proper development of the plants through the coordination of transcription and processing of a group of pri-miRNAs. In addition, CDF2 was found to regulate the expressions of components involved in miRNA biogenesis, therefore it may affect miRNA biogenesis through regulating the levels of these components. CDF2 was known to interact with blue light photoreceptor LKP2 and FKF1 and contribute to blue light signaling [18], it is therefore particularly interesting to study the roles of miRNAs in light signaling pathways which was implicated in a recent study showing that the E3 ligase COP1 essential for light signaling stabilizes HYL1 to retain miRNA biogenesis [39].

## Materials and Methods

### Plant materials

*Arabidopsis thaliana* (ecotype Col-0), *hyl1-2* (Salk_064863) [44], *dcl1-9* [45,46], *pDCL1-DCL1-YFP/dcl1-9* [27], *cdf2* [11], *miR156a*/*miR156c* [41] mutants, miR156 and miR172 overexpression lines [41] were used. All plants were grown in soil or Murashige and Skoog (MS) medium at 16 hr light/8 hr dark photoperiod.

### Constructs and transgenic plants

The cDNAs of CDF2 and DCL1 were cloned into pCambia1301 with an YFP epitope tag [27] and confirmed by sequencing. All binary vectors were introduced into *Agrobacterium tumefaciens* (GV3101) by electroporation. The floral dip method was used to transform the plants [47], and transgenic plants were selected on hygromycin (50mg/L). Transgenic lines with a single T-DNA insertion were selected according to the segregation ratio in the progeny of these transformants, and homozygous stocks were established from these lines. At least 15 T2 independent lines were analyzed for each construct.

### Yeast two-hybrid screen

The *Arabidopsis* cDNA library cloned in the prey vector pDEST22 was kindly provided by Dr. Jirong Huang. The two C-terminal double strand RNA binding domains of *Arabidopsis* DCL1 [48] were PCR-amplified and subcloned to the pDEST32 plasmid. Yeast transformation and library screening were performed according to the Pro-Quest Two-Hybrid System Manual (Matchmaker user’s manual, Invitrogen). Approximately 1.9 × 10^6^ yeast clones were screened on SC/-Leu-Trp-His medium with 5 mM 3-amino-1, 2, 4-triazole (3-AT). The β-galactosidase activity was analyzed in yeast strain MaV203, and the positive candidates were selected for sequencing.

In yeast two hybrid assays, different lengths of CDF2 C-terminal fragments were PCR-amplified and subcloned to the pDEST32 plasmid. The size of C1 fragment was 267 aa (from aa 189 to aa 457), C2 fragment was 177 aa (from aa 280 to aa 457), C3 fragment was 97 aa (from aa 360 to aa 457), C4 fragment was 75 aa (from aa 361 to aa 436), C5 fragment was 37 aa (from aa 361 to aa 398), C6 fragment was 61 aa (from aa 396 to aa 457), C7 fragment was 25 aa (from aa 396 to aa 421), and C8 fragment was 15 aa (from aa 385 to aa 400). Yeast co-transformation was performed according to the Pro-Quest Two-Hybrid System Manual (Matchmaker user’s manual, Invitrogen).

### Maltose-binding protein (MBP) and glutathione S-transferase (GST) pull-down assays

MBP-CDF2, MBP-DCL1-RBD and MBP were expressed in *E*. *coli* BL21 (DE3) and purified according to the manufacturer’s protocol (New England Biolabs). Glutathione–agarose 4B (Peptron) beads were used to purify GST-CDF2, GST-DCL1-RBD and GST. The partially purified recombinant GST and MBP fusion proteins were bounded to glutathione–agarose beads and amylose resin respectively, MBP-DCL1-RBD was incubated with the GST-CDF2 captured by glutathione sepharose beads, whereas GST-DCL1-RBD was incubated with the MBP-CDF2 bound to amylose resin. The GST and MBP proteins were performed in parallel assays as negative controls, mixed with total protein extracts in 1mL pull-down buffer (40 mM HEPES-KOH, pH 7.5, 10 mM KCl, 3 mM MgCl2, 0.4 M sucrose, 1 mM EDTA, 1 mM DTT, and 0.2% Triton X-100), and then incubated at 4°C for 1 h with agitation [49]. The beads were washed four times with the binding buffer. Proteins were eluted and further analyzed by immunoblotting using appropriate antibodies.

### Coimmunoprecipitation (Co-IP) assay

Plants coexpressing hemagglutinin (HA)-labeled CDF2 (*pCDF2*::*CDF2-HA)* and yellow fluorescent protein (YFP)-labeled DCL1 (*pDCL1*::*DCL1-YFP*) were generated by crossing *pDCL1*::*DCL1-YFP*/*dcl1-9* [26] with *pCDF2*::*CDF2-HA*/*cdf2* which was obtained by crossing *pCDF2*::*CDF2-HA*/Col with *cdf2* mutant. For a control, plants co-expressing *pCDF2*::*CDF2-HA/cdf2* and *p35S*::*YFP* were generated by transforming the *pCDF2*::*CDF2-HA/cdf2* line with the *p35*::*YFP* construct. The homozygous lines were grown in soil for 22 days, then seedlings were ground in liquid nitrogen and homogenized in three volumes of extraction buffer (50 mM Tris-HCl at pH 8.0, 150 mM NaCl, 0.5% TritonX-100, 0.2% 2-mercaptoethanol, 5% glycerol) containing complete proteinase inhibitor cocktail (Roche) using a mortar and pestle set [50]. Cell debris was pellet by centrifugation for 10 min at 15,000 rpm. The Co-IP experiments were performed using HA Tag IP/Co-IP Kit according to the manufacturer’s protocol (Thermo Pierce). Briefly, the mixed lysate was incubated with anti-HA agarose for 4 h to overnight in each spin column. The columns were washed five times with TBS plus 0.05% Tween-20 detergent (TBS-T), and resolved by SDS/PAGE. Anti-GFP (Sigma) and anti-HA (Cell signaling technology) antibodies were used to detect DCL1-YFP and CDF2-HA, respectively.

### Bimolecular Fluorescence Complementation (BiFC)

BiFC assays were perfomed as previously described [24]. The coding sequence of CDF2, DCL1, HYL1, DCL1-9 [24], the N-terminal fragment of CDF2 (CDF2-N, from aa 1 to 279), and the C-terminal fragment of CDF2 (CDF2-C, from aa 280 to 457) were subcloned into pCambia1301 vectors with a YFPN or YFPC tag [24] and confirmed by sequencing. Primers used to amplify these genes are listed in S3 Table. Paired YFPN and YFPC-tagged plasmids were co-expressed in tobacco leaves. 48 hours after co-inoculation, BiFC signals were visualized with a DeltaVision PersonalDV system (Applied Precision) using an Olympus UPLANAPO waterimmersion objective lens (60×/1.20 numerical aperture).

### Real-time quantitative PCR

Total RNA was extracted from *Arabidopsis* seedlings using Trizol. The first strand cDNA was generated from the total RNA (5 μg) using M-MLV reverse transcriptase (Promega) and used as the template for subsequent PCR. The real-time quantitative PCR (RT-qPCR) for examination of pri-miRNAs expression was carried out with a BIO-RAD CFXTM Real-Time System. The *actin* gene was used as internal control for normalization of the template cDNA. Each PCR was repeated at least three times. The PCR thermal cycles were as follows: an initial denature step consisting of 2 min at 50°C and 10 min at 95°C, followed by 45 cycles of 30 s at 94°C, 30 s at 52°C and 18 s at 72°C, and an additional cycle of 15 s at 95°C, 15 s at 60°C and 15 s at 95°C for melting curve analysis [51]. The data were analyzed with a Bio-Rad iCycler iQ Real-Time Detection System. The relative expressions of pri-miRNAs was calculated using the relative 2^–ΔΔCt^ method [52] and the error bars indicate SD (n = 3).

### Chromatin immunoprecipitation (ChIP)

The ChIP experiments were performed as described [53] using 22-day-old seedlings. *pCDF*::CDF2-YFP transgenic seedlings were harvested in cross-linking buffer (0.4M sucrose, 10mM Tris-HCL(pH8.0), 1mM PMSF, ImM EDTA, 1% formaldehyde) for 10 min using vacuum infiltration and then stopped in 2M glycine. After chromatin shearing, 5-μg anti-GFP antibody (Sigma, G6495) was added to the samples and incubated at 4°C overnight, then washed and eluted the beads with the lysis buffer (0.1M NaHCO_3_, 1%SDS). After reversing the cross-linking, DNA was precipitated using ethanol, and resuspended in 50 ul water. The amounts of the immunoprecipitated genomic DNA were used for PCR and quantified by real-time PCR, *actin* gene is used as internal control. Primers used to amplify the promoters of some miRgenes are specified in S3 Table.

### Small RNA gel blot

Small RNAs were isolated from seedlings of 22-day-old plants or inflorescences using mirVana™miRNA Isolation Kit (Ambion, AM1561). Three micrograms of small RNAs were separated on a 15% polyacrylamide gel with 8 M urea and transferred to a nylon transfer membrane (GE Healthcare). The antisense oligonucletides (S3 Table) were synthesized and 3’end-labeled with biotin. Hybridization was performed overnight in hybridization buffer (Ambion, AM8663) at 42°C. A probe complementary to U6 (5’CATCCTTGCGCAGGGGCCA 3’) was used as a loading control.

### Histochemical GUS staining

GUS staining was performed according to the standard procedure [54] with a modified buffer(1 mg/mL 5-bromo-4-chloro-3-indolyl-b-D-glucuronic acid cyclohexylammonium salt, 50 mM sodium phosphate, pH 7.0, 0.1% Triton X-100, 2 mM potassium ferrocyanide, 2 mM potassium ferricyanide, and 10 mM EDTA). Plant tissues were incubated in the buffer at 37°C in the dark overnight, then cleared with 75% ethanol, followed by observation [19].

### RNA electrophoretic mobility shift assay (RNA EMSA)

Pri-miR167b fragment was amplified from Col plant cDNAs using specific primers with a T7 promoter sequence fused to the 5’ terminal of the forward primers (S3 Table). The PCR products were then used as templates for in vitro transcription (Megascript; Ambion), then make biotin labeled or unlabeled RNA transcripts using RNA 3’ End Biotinylation Kit (Thermo Pierce). Using LightShift Chemiluminescent RNA EMSA Kit (Thermo Pierce) according to manufactory’s protocol RNA EMSA assays were performed in a 20-μl reaction buffer containing 5% glycerol, 2ug tRNA and 1 nM of labeled pri-miR167b transcripts and purified recombinant proteins, including CDF2, DCL1-RBD, HYL1, CDF2-N (from aa 1 to 279) and CDF2-C (from aa 280 to 457). The mixtures were incubated on ice for 30 min and then fractioned on a 6% native polyacrylamide gel in 1x TBE buffer for about 60 min. The gels were transferred to a nylon membrane (GE Healthcare) and then the biotin-labeled RNAs were detected by Chemiluminescence (Thermo Pierce) [10].

In the assay of examining the competition ability of CDF2 with DCL1 in their binding to pri-miRNA167 (Figs 4A and S7A–S7D), recombinant proteins and biotin-labeled 1nM pri-miR167b which were added in all lanes, unlabeled pri-miR167b was added in lane 8–10. Lane 1, 5μg DCL1-RBD; lane 2, 5μg CDF2; lane 3, 5μg DCL1-RBD+ 1μg CDF2; lane 4, 5μg DCL1-RBD+ 2μg CDF2; lane 5, 5μg DCL1-RBD+ 3μg CDF2; lane 6, 5μg DCL1-RBD+ 4μg CDF2; lane 7, 5μg DCL1-RBD+ 5μg CDF2; lane 8, 5μg DCL1-RBD+ 2μM unlabled pri-miR167b; lane 9, 5μg DCL1-RBD+ 1μM unlabeled pri-miR167b; lane 10, 5μg DCL1-RBD+ 0.5μM unlabeled pri-miR167b.

In the assay to examine the roles of CDF2 and DCL1-RBD in their bindings to pri-miRNA167 (S7E Fig), recombinant proteins and biotin-labeled 1nM pri-miR167b which were added in all lanes. Lane 1, 5μg DCL1-RBD; lane 2, 5μgCDF2; lane 3, 5μgC-terminal fragment of CDF2 (CDF2-C); lane 4, 5μg N-terminal fragment of CDF2 (CDF2-N).

### Small RNA deep sequencing

Small RNAs were isolated from seedlings of 22-day-old plants using mirVana miRNA Isolation Kit (Ambion, AM1561). Small RNA libraries were prepared and sequenced by Illumina Solexa high-throughput sequencing. The small RNA reads were trimmed for adaptor sequence using Perl scripts and mapped to miRNA hairpin sequences downloaded from mirRBase version 15.0 using the Bowtie program [55]. The total numbers of perfectly aligned reads were used for normalization. After removing the linker sequence, reads were aligned to the miRNA database (version 16) [56] to identify and assess the abundance of known miRNAs. The sequencing data for all known miRNAs were subjected to hierarchical clustering in an unsupervised manner to analyze the extent of differential miRNA expression.

### RNA immunoprecipitation (RIP) assay

The RIP assay was performed as described [57] with some modifications. Briefly, 5 to 10 g of 22-day-old plants of *pCDF2*::CDF2-YFP/Col, *pDCL1*::DCL1-YFP/Col (as a positive control), *pDCL1*::*DCL1-YFP*/*cdf2*, and *pDCL1*::*DCL1-YFP*/Col crossed by *p35S*::*CDF2-HA*, wild-type (as a negative control) plants were fixed. After sonication. the total soluble nuclear proteins were extracted. A portion of each nuclear extract was immunoprecipitated with the RNA-Binding Protein Immunoprecipitation Kit according to the manufacturer’s protocol (Millipore), using Anti-GFP (Sigma) antibody-coupled magnetic A/G beads or IgG coupled magnetic protein A/G beads. 100μl of each nuclear protein was stored at -70°C for input preparation. RNAs were extracted from the immunoprecipitated products and each input. The resulting cDNAs from these RNAs were used for RT-PCR analysis, *actin* gene is used as an internal control.

## Supporting Information

S1 Fig The purified proteins resolved on a SDS polyacrylamide gel and detected by Coomassie light blue staining. (TIF)

S2 Fig The expression levels of transgenes and phenotypes ofthe transgenic plants indicated. (A) The relative expression levels of *CDF2* and *DCL1* in *pCDF2*::CDF2-*HA/YFP* and *pDCL1*::*DCL1-YFP Arabidopsis* transgenic lines compared to corresponding genes in Col. Data are given as means ± SD (n = 3). (B) 24-day-old plants of indicated genotypes grown in long day photoperiods (16 hours light, 8 hours dark). (C) The time of flowering of the indicated genotypes shown in B. Data are mean ± SEM of 50 plants. (D) The number of rosette leaves at the time of flowering of the indicated genotypes shown in B. Data are means ± SEM of 50 plants.
(TIF)

S3 Fig Yeast two hybrid assays show that a C terminal fragment of CDF2 interacts with DCL1-RBD. C4, aa 361–436; C5, aa 361–398; C6, aa 396–457; C7, aa 396–421, C8, aa 385–400.
(TIF)

S4 Fig Comparision of miRNAs that are differently expressed in Col and *dcl1-9* mutant. The plants were grown for 22 days before tissues were collected for RNA extraction. Small RNA was isolated and sequenced by Illumina high-throughput sequencing.
(TIF)

S5 Fig Northen blots of miRNAs in 22-day-old seedlings and inflorescences of Col and *cdf2*. (A) Northern blots show the levels of miRNAs in 22-day-old seedlings of Col and *cdf2* mutant. U6 serves as a loading control. (B) Northern blots show the levels of miRNAs in inflorescences of Col and *cdf2* mutant. U6 serves as a loading control.
(TIF)

S6 Fig The relative expression levels of *DCL1* and *HYL1* in *p35S*::*CDF2-YFP/Col*, and *cdf2* mutant compared to corresponding genes in Col. Data are given as means ± SD (n = 3).
(TIF)

S7 Fig The different effects of CDF2 on the bindings of DCL1-RBD to pri-miRNA167. (A)-(C) RNA EMSAs show the effect of CDF2 on the binding of DCL1-RBD to pri-miRNA167. (D) The quantitative analysis of RNA binding activities of Lane1-7 in Figs 4A and S7A-S7C. (E) RNA EMSAs show the binding of CDF2 to pri-miRNA167 is mainly mediated by its C-terminal fragment.
(TIF)

S8 Fig Western blot analysis of the immunoprecipitated samples used for RIP assays. RIP assays were performed using 22-day-old plants, RNA fragments were immunoprecipitated with a GFP antibody. The CDF2-YFP and DCL1-YFP proteins were detected by an anti-GFP antibody.“*” stands for a nonspecific signal.
(TIF)

S9 Fig The relative levels of pri-miRNAs in Col, *hyl1*, *cdf2*, and *hyl1/cdf2* lines examined by real-time PCR. The relative fold changes were normalized to *ACTIN*. Data are given as means ± SD (n = 3).
(TIF)

S10 Fig The characterization of plants transformed with *p35S*::*CDF2-YFP*, *p35S*::*DCL1-YFP* and *pCDF2*::*CDF2-YFP*. (A) The relative expression levels of *CDF2*, *DCL1* and *CDF2* in *p35S*::*CDF2-YFP*, *p35S*::*DCL1-YFP* and *pCDF2*::*CDF2-YFP* transgenic lines compared to corresponding genes in Col. Data are given as means ± SD (n = 3). (B) Northern blots show that the CDF2 transgenic lines driven by its endogenous promoter do not affect the miRNA accumulation.
(TIF)

S11 Fig The characterization of miR156 and miR172 overexpression lines in Col and *cdf2* backgrounds. The relative expression levels of *miR156* and *miR172* in Col, *p35S*::miR156/Col and *p35S*::miR156/*cdf2* or *p35S*::miR172/Col and *p35S*::miR172/*cdf2* lines were detected by qRT-PCR. Data are given as means ± SD (n = 3).
(TIF)

S12 Fig The effect of CDF2 on the expressions of components involved in miRNA biogenesis. The relative expression levels of *DCL1*, *HYL1*, *SE*, *MOS2*, *CDC5*, *TOUGH*, *DDL*, *NOT2*, *CPL1* were detected by qRT-PCR in Col wild type, *cdf2* mutant and *CDF2* overexpression lines. Data are given as means ± SD (n = 3).
(TIF)

S13 Fig The expression levels of miR156 and miR172 targeted genes. (A) The relative expressional levels of *SPLs* in Col, *cdf2* mutant, *p35S*::miR156/Col and *p35S*::miR156/*cdf2*. (B) The relative expressional levels of *AP2* in Col, *cdf2* mutant, *p35S*::miR172/Col and *p35S*::miR172/*cdf2*. Data are given as means ± SD (n = 3).
(TIF)

S14 Fig Phenotypes of Col, *cdf2* and *hyl1* single mutants, and *cdf2/hyl1* double mutants. (A) 24-day-old plants of indicated genotypes grown in long day photoperiods (16 hours light, 8 hours dark). (B) The flowering time of the indicated genotypes shown in A. Data are mean ± SEM of 50 plants. (C) The number of rosette leaves at the time of flowering of the indicated genotypes shown in A. Data are means ± SEM of 50 plants.
(TIF)

S1 Table The accumulation levels of miRNAs changed more than 1.5 fold in *cdf2* mutant. (DOC)

S2 Table The accumulation levels of miRNAs changed more than 1.5 fold in *dcl1-9* mutant (DOC)

S3 Table Oligonucleotide primer sequence Figure Legends (DOC)

## References

[1] Vazquez F, Legrand S, Windels D (2010) The biosynthetic pathways and biological scopes of plant small RNAs. Trends in Plant Science 15: 337–345. 10.1016/j.tplants.2010.04.001 20427224

[2] Chapman E, Carrington J (2007) Specialization and evolution of endogenous small RNA pathways. Nature Reviews Genetics 8: 884–896. 17943195 10.1038/nrg2179

[3] Yu B, Bi L, Zheng B, Ji L, Chevalier D, et al (2008) The FHA domain proteins DAWDLE in Arabidopsis and SNIP1 in humans act in small RNA biogenesis. Proc Natl Acad Sci U S A 105: 10073–10078. 10.1073/pnas.0804218105 18632581 PMC2481372

[4] Jones-Rhoades M, Bartel D, Bartel B (2006) MicroRNAs and Their Regulatory Roles in Plants. Annu Rev Plant Biol 57: 19–53. 16669754 10.1146/annurev.arplant.57.032905.105218

[5] Han M, Goud S, Song L, Fedoroff N (2004) The Arabidopsis double-stranded RNA-binding protein HYL1 plays a role in microRNA-mediated gene regulation. Proceedings of the National Academy of Sciences 101: 1093–1098. 10.1073/pnas.0307969100 PMC327156 14722360

[6] Lobbes D, Rallapalli G, Schmidt D, Martin C, Clarke J (2006) SERRATE: a new player on the plant microRNA scene. EMBO reports 7: 1052–1058. 16977334 10.1038/sj.embor.7400806 PMC1618363

[7] Manavella P, Hagmann J, Ott F, Laubinger S, Franz M, et al (2012) Fast-Forward Genetics Identifies Plant CPL Phosphatases as Regulators of miRNA Processing Factor HYL1. Cell 151: 859–870. 10.1016/j.cell.2012.09.039 23141542

[8] Laubinger S, Sachsenberg T, Zeller G, Busch W, Lohmann JU, et al (2008) Dual roles of the nuclear cap-binding complex and SERRATE in pre-mRNA splicing and microRNA processing in Arabidopsis thaliana. Proc Natl Acad Sci U S A 105: 8795–8800. 10.1073/pnas.0802493105 18550839 PMC2426964

[9] Ren G, Xie M, Dou Y, Zhang S, Zhang C, et al (2012) Regulation of miRNA abundance by RNA binding protein TOUGH in Arabidopsis. Proc Natl Acad Sci U S A 109: 12817–12821. 10.1073/pnas.1204915109 22802657 PMC3412041

[10] Wu X, Shi Y, Li J, Xu L, Fang Y, et al (2013) A role for the RNA-binding protein MOS2 in microRNA maturation in Arabidopsis. Cell Res 23: 645–657. 10.1038/cr.2013.23 23399598 PMC3641593

[11] Fornara F, Panigrahi K, Gissot L, Sauerbrunn N, Rühl M, et al (2009) Arabidopsis DOF Transcription Factors Act Redundantly to Reduce CONSTANS Expression and Are Essential for a Photoperiodic Flowering Response. Developmental Cell 17: 75–86. 10.1016/j.devcel.2009.06.015 19619493

[12] Moreno-Risueno M, Martínez M, Vicente-Carbajosa J, Carbonero P (2007) The family of DOF transcription factors: from green unicellular algae to vascular plants. Molecular Genetics and Genomics 277: 379–390. 17180359 10.1007/s00438-006-0186-9

[13] Papi M, Sabatini S, Bouchez D, Camilleri C, Costantino P, et al (2000) Identification and disruption of an Arabidopsis zinc finger gene controlling seed germination. Genes Development 14: 28–33. 10640273 PMC316352

[14] Gualberti G, Papi M, Bellucci L, Ricci I, Bouchez D, et al (2002) Mutations in the Dof Zinc Finger Genes DAG2 and DAG1 Influence with Opposite Effects the Germination of Arabidopsis Seeds. The Plant Cell Online 14: 1253–1263. 10.1105/tpc.010491 PMC150778 12084825

[15] Kang H, Singh K (2000) Characterization of salicylic acid-responsive, arabidopsis Dof domain proteins: overexpression of OBP3 leads to growth defects. The Plant Journal 21: 329–339. 10758484 10.1046/j.1365-313x.2000.00678.x

[16] Park D, Lim P, Kim J, Cho D, Hong S, et al (2003) The Arabidopsis COG1 gene encodes a Dof domain transcription factor and negatively regulates phytochrome signaling. The Plant Journal 34: 161–171. 12694592 10.1046/j.1365-313x.2003.01710.x

[17] Peng Z, Lu Y, Li L, Zhao Q, Feng Q, et al (2013) The draft genome of the fast-growing non-timber forest species moso bamboo (Phyllostachys heterocycla) Nat Genet 45: 456–461. 10.1038/ng.2569 23435089

[18] Imaizumi T, Schultz TF, Harmon FG, Ho LA, SA. K (2005) FKF1 F-box protein mediates cyclic degradation of a repressor of CONSTANS in Arabidopsis. Science 309: 293–297. 16002617 10.1126/science.1110586

[19] Wang L, Song X, Gu L, Li X, Cao S, et al (2013) NOT2 proteins promote polymerase II-dependent transcription and interact with multiple MicroRNA biogenesis factors in Arabidopsis. Plant Cell 25: 715–727. 10.1105/tpc.112.105882 23424246 PMC3608788

[20] Zhang S XM, Ren G, Yu B. (2013) CDC5, a DNA binding protein, positively regulates posttranscriptional processing and/or transcription of primary microRNA transcripts. Proc Natl Acad Sci U S A 110: 17588–17593. 10.1073/pnas.1310644110 24101471 PMC3808604

[21] Cheng TL WZ, Liao Q, Zhu Y, Zhou WH, Xu W, Qiu Z. (2014) MeCP2 suppresses nuclear microRNA processing and dendritic growth by regulating the DGCR8/Drosha complex. Dev Cell 28: 547–560. 10.1016/j.devcel.2014.01.032 24636259

[22] Wagschal A, Rousset E, Basavarajaiah P, Contreras X, Harwig A, et al (2012) Microprocessor, Setx, Xrn2, and Rrp6 co-operate to induce premature termination of transcription by RNAPII. cell 150: 1147–1157. 10.1016/j.cell.2012.08.004 22980978 PMC3595997

[23] Liu F, Bakht S, C. D (2012) Cotranscriptional role for Arabidopsis DICER-LIKE 4 in transcription termination. science 335: 1621–1623. 10.1126/science.1214402 22461611

[24] Fang Y, Spector D (2007) Identification of Nuclear Dicing Bodies Containing Proteins for MicroRNA Biogenesis in Living Arabidopsis Plants Curr Biol 17: 818–823. 17442570 10.1016/j.cub.2007.04.005 PMC1950788

[25] Liu Q, Yan Q, Liu Y, Hong F, Sun Z, et al (2013) Complementation of HYPONASTIC LEAVES1 by double-strand RNA-binding domains of DICER-LIKE1 in nuclear dicing bodies. Plant Physiol 163: 108–117. 10.1104/pp.113.219071 23886622 PMC3762634

[26] Fang Y, DL S (2007) Identification of nuclear dicing bodies containing proteins for microRNA biogenesis in living Arabidopsis plants. Curr Biol 17: 818–823. 17442570 10.1016/j.cub.2007.04.005 PMC1950788

[27] Eisen M, Spellman P, Brown P, D B (1998) Cluster analysis and display of genome-wide expression patterns. Proc Natl Acad Sci 95: 14863–14868. 9843981 10.1073/pnas.95.25.14863 PMC24541

[28] Yanagisawa S (2002) The Dof family of plant transcription factors. TRENDS in Plant Science 7: 555–560. 12475498 10.1016/s1360-1385(02)02362-2

[29] Mena M, Cejudo F, Isabel-Lamoneda I, Carbonero P (2002) A Role for the DOF Transcription Factor BPBF in the Regulation of Gibberellin-Responsive Genes in Barley Aleurone. Plant Physiology 130: 111–119. 12226491 10.1104/pp.005561 PMC166544

[30] Fang X, Cui Y, Li Y, Qi Y. (2015) Transcription and processing of primary microRNAs are coupled by Elongator complex in Arabidopsis. NATURE PLANTS 10.1038/nplants.2015.75 27250010

[31] Kawahara Y, Zinshteyn B, Sethupathy P, Iizasa H, Hatzigeorgiou AG, et al (2007) Science. Redirection of silencing targets by adenosine-to-inosine editing of miRNAs 315: 1137–1140. 17322061 10.1126/science.1138050 PMC2953418

[32] Yamagata K, Fujiyama S, Ito S, Ueda T, Murata T, et al (2009) Maturation of MicroRNA Is Hormonally Regulated by a Nuclear Receptor. Molecular Cell 36: 340–347. 10.1016/j.molcel.2009.08.017 19854141

[33] Heo I, Joo C, Kim YK, Ha M, Yoon MJ, et al (2009) TUT4 in concert with Lin28 suppresses microRNA biogenesis through pre-microRNA uridylation. Cell 138: 696–708. 10.1016/j.cell.2009.08.002 19703396

[34] Rybak A, Fuchs H, Smirnova L, Brandt C, Pohl EE, et al (2008) A feedback loop comprising lin-28 and let-7 controls pre-let-7 maturation during neural stem-cell commitment. Nat Cell Biol 10: 987–993. 10.1038/ncb1759 18604195

[35] Viswanathan SR, Daley GQ, RI. G (2008) Selective blockade of microRNA processing by Lin28. Science 320: 97–100 10.1126/science.1154040 18292307 PMC3368499

[36] Heo I, Joo C, Kim Y, Ha M, Yoon M, et al (2009) TUT4 in Concert with Lin28 Suppresses MicroRNA Biogenesis through Pre-MicroRNA Uridylation. Cell 138: 696–708. 10.1016/j.cell.2009.08.002 19703396

[37] Hagan J, Piskounova E, Gregory R (2009) Lin28 recruits the TUTase Zcchc11 to inhibit let-7 maturation in mouse embryonic stem cells. Nat Struct Mol Biol 16: 1021–1025. 10.1038/nsmb.1676 19713958 PMC2758923

[38] Luo Y, Guo Z, L. L (2013) Evolutionary conservation of microRNA regulatory programs in plant flower development. Dev Biol 380: 133–144. 10.1016/j.ydbio.2013.05.009 23707900

[39] Cho SK, Chaabane SB, Shah P, Poulsen CP, SW. Y (2014) COP1 E3 ligase protects HYL1 to retain microRNA biogenesis. Nat Commun 23: 10.1038/ncomms6867 25532508

[40] Wang JW, Czech B, W D (2009) miR156-regulated SPL transcription factors define an endogenous flowering pathway in Arabidopsis thaliana. Cell 138: 738–749 10.1016/j.cell.2009.06.014 19703399

[41] Wang JW, Czech B, D. W (2009) miR156-regulated SPL transcription factors define an endogenous flowering pathway in Arabidopsis thaliana. Cell 138: 738–749. 10.1016/j.cell.2009.06.014 19703399

[42] Yang Z, Wang X, Gu S, Hu Z, Xu H, et al (2008) Comparative study of SBP-box gene family in Arabidopsis and rice. Gene 407: 1–11. 17629421 10.1016/j.gene.2007.02.034

[43] Chen X (2004) A microRNA as a translational repressor of APETALA2 in Arabidopsis flower development Science 303: 2022–2025. 12893888 10.1126/science.1088060 PMC5127708

[44] Lu C, Fedoroff NV (2000) A mutation in the Arabidopsis HYL1 gene encoding a dsRNA binding protein affects responses to abscisic acid, auxin, and cytokinin. Plant Cell 12: 2351–2366. 11148283 10.1105/tpc.12.12.2351 PMC102223

[45] Vazquez F, Vaucheret H, Rajagopalan R, Lepers C, Gasciolli V, et al (2004) Endogenous trans-acting siRNAs regulate the accumulation of Arabidopsis mRNAs. Mol Cell 16: 69–79. 15469823 10.1016/j.molcel.2004.09.028

[46] Jacobsen S, Running M, Meyerowitz E (1999) Disruption of an RNA helicase/RNAse III gene in Arabidopsis causes unregulated cell division in floral meristems. Development 126: 5231–5243. 10556049 10.1242/dev.126.23.5231

[47] Clough S, Bent A (1998) Floral dip: a simplified method for Agrobacterium-mediated transformation of Arabidopsis thaliana. Plant J 16: 735–743. 10069079 10.1046/j.1365-313x.1998.00343.x

[48] Liu Q, Yan Q, Liu Y, Hong F, Sun Z, et al (2013) Complementation of HYPONASTIC LEAVES1 by double-strand RNA-binding domains of DICER-LIKE1 in nuclear dicing bodies. Plant Physiol 163: 108–117. 10.1104/pp.113.219071 23886622 PMC3762634

[49] Ning J, Zhang B, Wang N, Zhou Y, L. X (2011) Increased leaf angle1, a Raf-like MAPKKK that interacts with a nuclear protein family, regulates mechanical tissue formation in the Lamina joint of rice. Plant Cell 23: 4334–4347. 10.1105/tpc.111.093419 22207574 PMC3269869

[50] Siurkus J, Panula-Perälä J, Horn U, Kraft M, Rimseliene R, et al (2010) Novel approach of high cell density recombinant bioprocess development: optimisation and scale-up from microliter to pilot scales while maintaining the fed-batch cultivation mode of E. coli cultures. Microb Cell Fact 9. 10.1186/1475-2859-9-35 PMC2890543 20487563

[51] Wang M, Gu D, Liu T, Wang Z, Guo X, et al (2007) Overexpression of a putative maize calcineurin B-like protein in Arabidopsis confers salt tolerance. Plant Mol Biol 65: 733–746. 17882512 10.1007/s11103-007-9238-8

[52] Livak KJS, Thomas D. (2001) Analysis of Relative Gene Expression Data Using Real-Time Quantitative PCR and the 2−ΔΔCT Method. Methods 25: 402–408. 11846609 10.1006/meth.2001.1262

[53] Saleh A, Alvarez-Venegas R, Z. A (2008) An efficient chromatin immunoprecipitation (ChIP) protocol for studying histone modifications in Arabidopsis plants. Nat Protoc 3: 1018–1025. 10.1038/nprot.2008.66 18536649

[54] Jefferson R.A., Kavanagh T.A., and Bevan MW (1987) GUS fusions: Beta-glucuronidase as a sensitive and versatile gene fusion marker in higher plants. EMBO J 6: 3901–3907. 3327686 10.1002/j.1460-2075.1987.tb02730.x PMC553867

[55] Langmead B, Trapnell C, Pop M, Salzberg S (2009) Ultrafast and memory-efficient alignment of short DNA sequences to the human genome. Genome Biology 10: R25 10.1186/gb-2009-10-3-r25 19261174 PMC2690996

[56] Griffiths-Jones S, Saini H, van Dongen S, Enright A (2008) miRBase: tools for microRNA genomics. Nucleic Acids Research 36: D154–D158. 17991681 10.1093/nar/gkm952 PMC2238936

[57] Terzi LC, and Simpson GG (2009) Arabidopsis RNA immunoprecipitation. Plant J 59: 163–168. 10.1111/j.1365-313X.2009.03859.x 19419533

