# Correction: The Roles of *Arabidopsis* CDF2 in Transcriptional and Posttranscriptional Regulation of Primary MicroRNAs

**DOI:** 10.1371/journal.pgen.1005700

**Published:** 2015-11-19

**Authors:** Zhenfei Sun, Tongtong Guo, Yin Liu, Qi Liu, Yuda Fang

There is an error in the last paragraph of the subsection ‘CDF2 interacts with DCL1’ in the Results and Discussion. “The results showed that the C-terminal fragments of CDF2 interacted with DCL1 as strongly as the full-length CDF2 (Fig 1D),” should read “The results showed that the C-terminal fragments of CDF2 interacted with DCL1 as strongly as the full-length CDF2 (Fig 1E).”

There is an error in the caption for Fig 3. “(B) ChIP-PCR analysis of five promoter fragments of miRNA genes,” should read “(B) ChIP-PCR analysis of six promoter fragments of miRNA genes”. Please see the complete, correct Fig 3 caption here.

**Fig 3:**
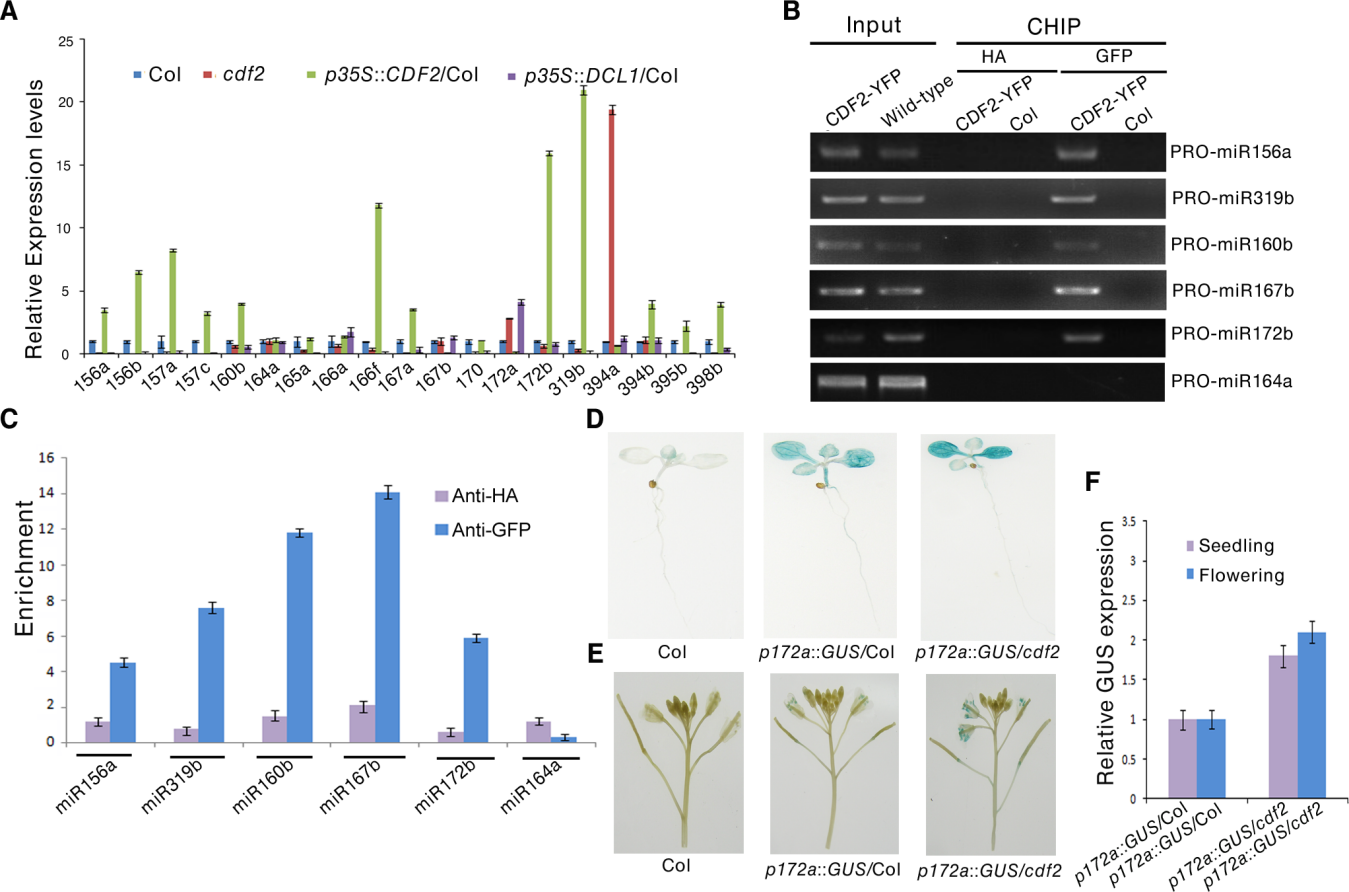
CDF2 acts as a transcription factor for some miRNA genes. (A) The relative levels of pri-miRNAs in Col, cdf2, p35S::CDF2/Col, p35S::DCL1/Col lines examined by real-time PCR. The relative fold changes were normalized to ACTIN. Data are given as means ± SD (n = 3). (B) ChIP-PCR analysis of six promoter fragments of miRNA genes in wild-type and pCDF2:CDF2-YFP/Col seedlings. ChIP assays were performed using the 22-day-old Col-0 and pCDF2::CDF2-YFP/Col seedlings expressing the CDF2-YFP fusion protein. DNA was amplified using primers specific to 6 miRNA promoter regions. (C) CHIP followed by real time PCR of 6 promoter fragments of miRNA genes in Col and pCDF2::CDF2-YFP/Col seedlings. Relative enrichment of fragments was calculated with HA antibodies as the control. Data are given as means ± SD (n = 3). (D) and (E) pMIR172a::GUS in Col, cdf2 and p35S::DCL1/Col in seedlings (D) and flowers (E), respectively. Thirty plants containing GUS were analyzed for each of genotypes. (F) The transcript levels of GUS driven by miR172b promoter in Col, cdf2 and p35S::DCL1/Col. GUS transcript levels were determined by qRT-PCR. Data are given as means ± SD (n = 3).
