# Supplementary figures and images for: The Roles of Arabidopsis CDF2 in Transcriptional and Posttranscriptional Regulation of Primary MicroRNAs
Source: PLoS Genet. 2015 Oct 16;11(10):e1005598. doi: 10.1371/journal.pgen.1005598 (PMC4608766; doi:10.1371/journal.pgen.1005598)

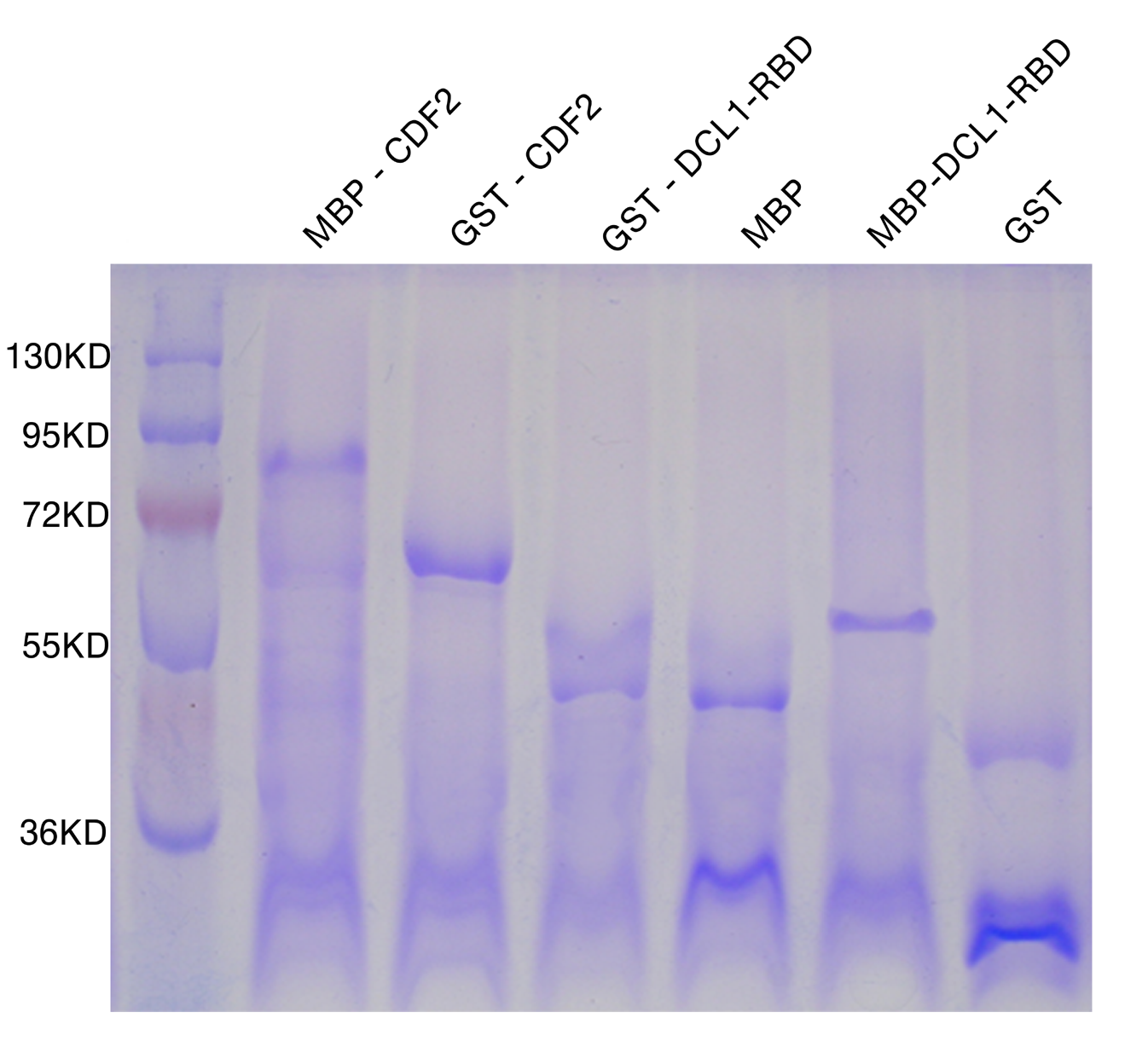

Supplement: S1 Fig — (TIF) [file pgen.1005598.s001.tif]

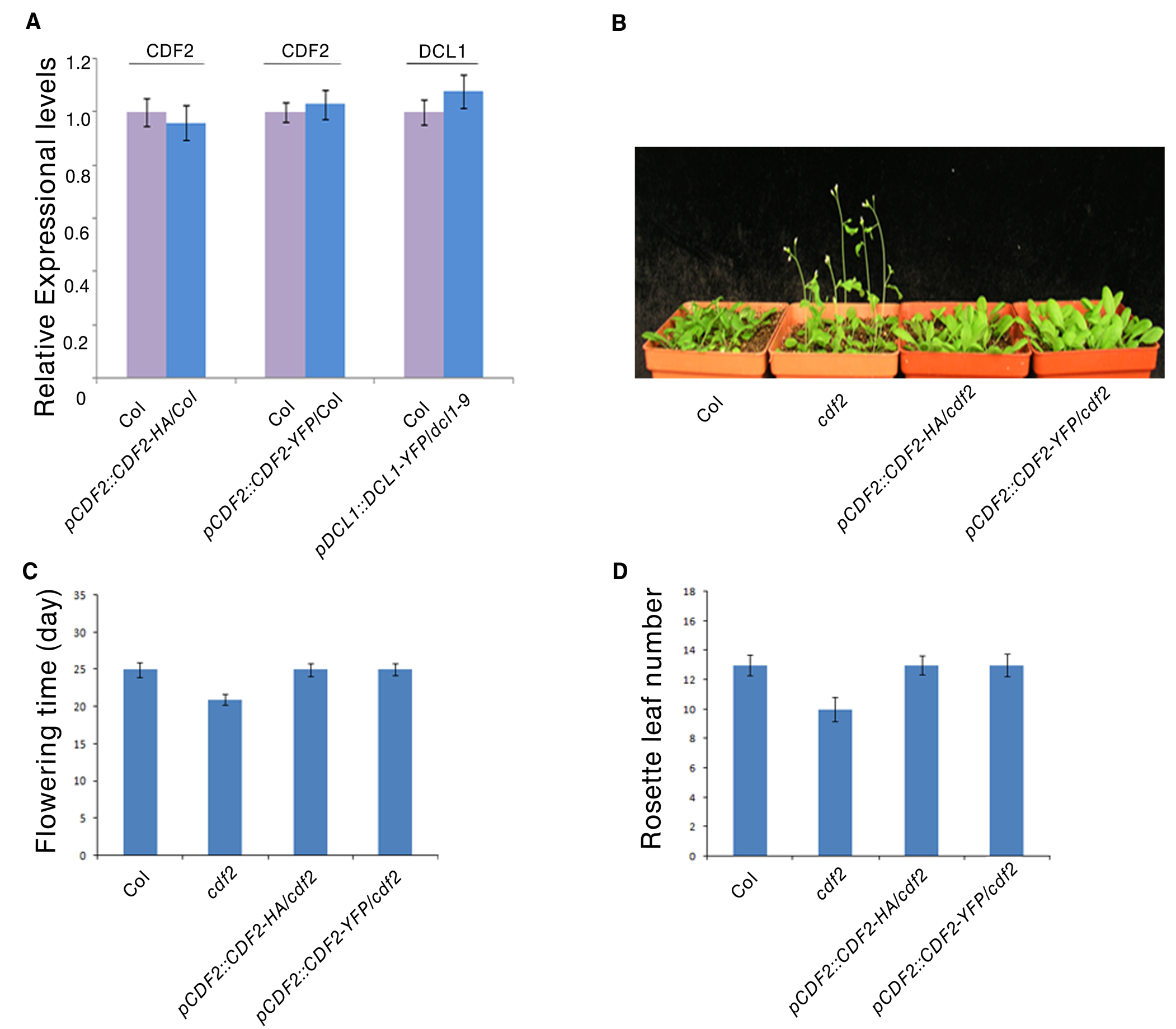

Supplement: S2 Fig — (A) The relative expression levels of CDF2 and DCL1 in pCDF2::CDF2-HA/YFP and pDCL1::DCL1-YFP Arabidopsis transgenic lines compared to corresponding genes in Col. Data are given as means ± SD (n = 3). (B) 24-day-old plants of indicated genotypes grown in long day photoperiods (16 hours light, 8 hours dark). (C) The time of flowering of the indicated genotypes shown in B. Data are mean ± SEM of 50 plants. (D) The number of rosette leaves at the time of flowering of the indicated genotypes shown in B. Data are means ± SEM of 50 plants. (TIF) [file pgen.1005598.s002.tif]

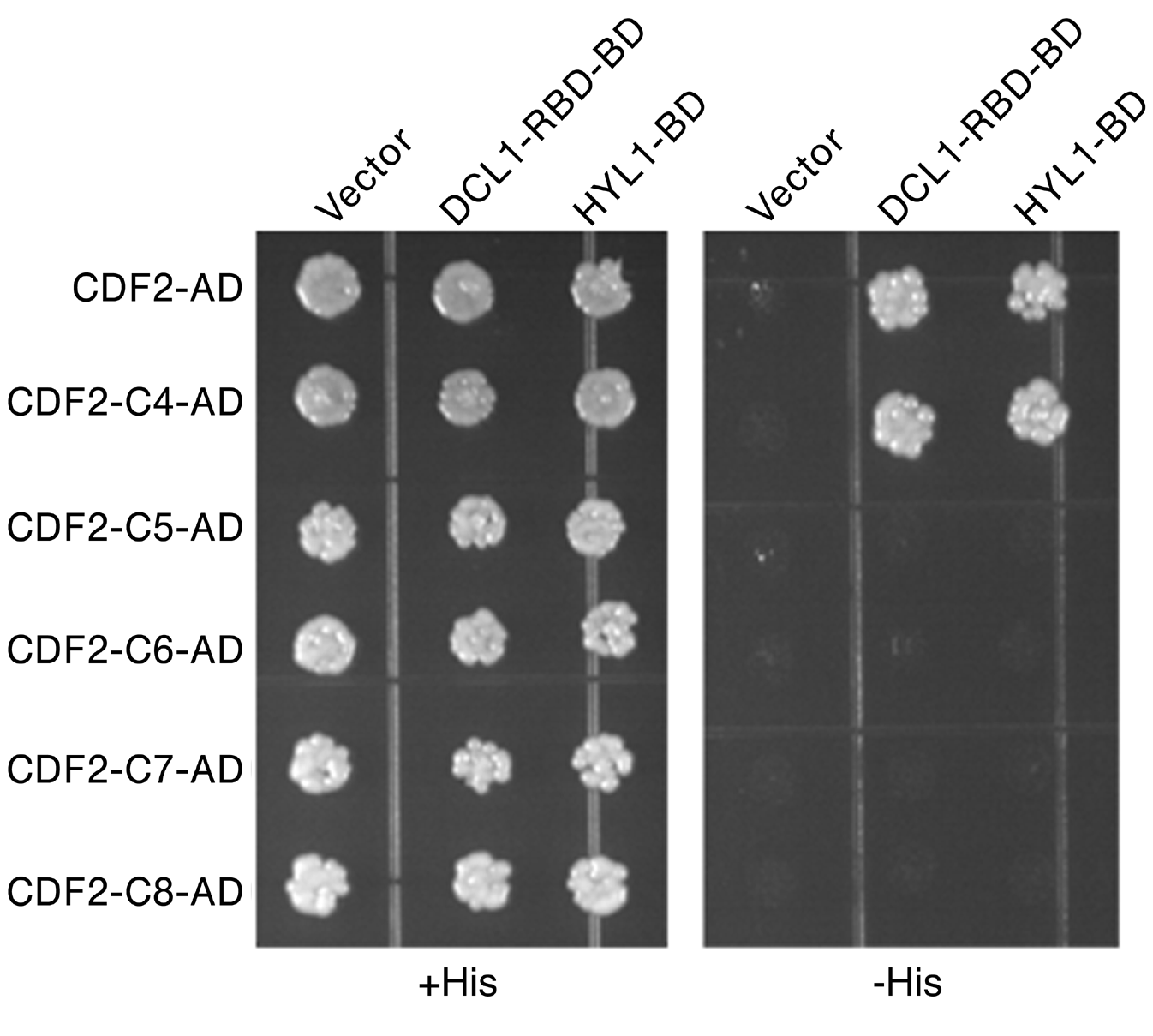

Supplement: S3 Fig — C4, aa 361–436; C5, aa 361–398; C6, aa 396–457; C7, aa 396–421, C8, aa 385–400. (TIF) [file pgen.1005598.s003.tif]

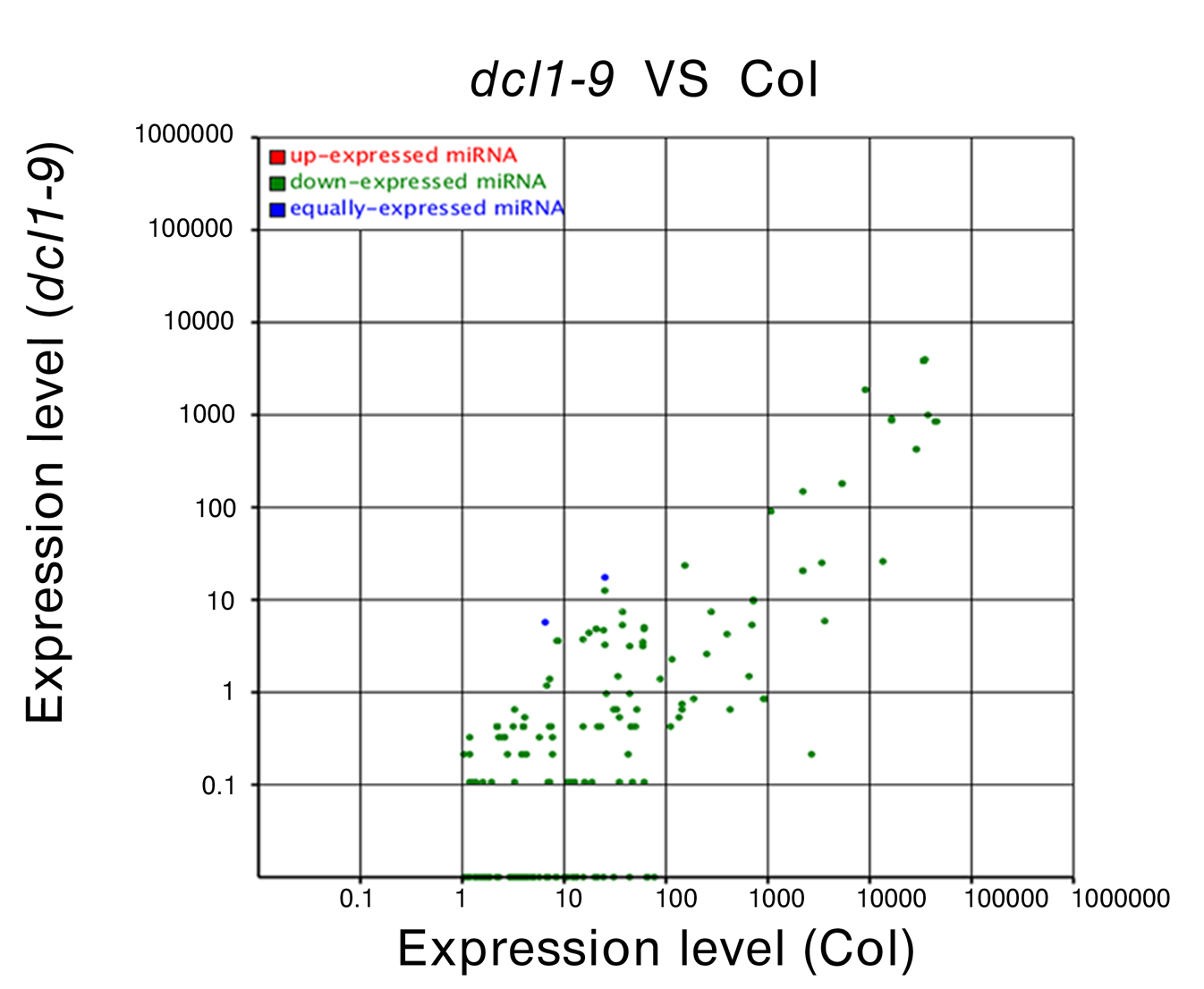

Supplement: S4 Fig — The plants were grown for 22 days before tissues were collected for RNA extraction. Small RNA was isolated and sequenced by Illumina high-throughput sequencing. (TIF) [file pgen.1005598.s004.tif]

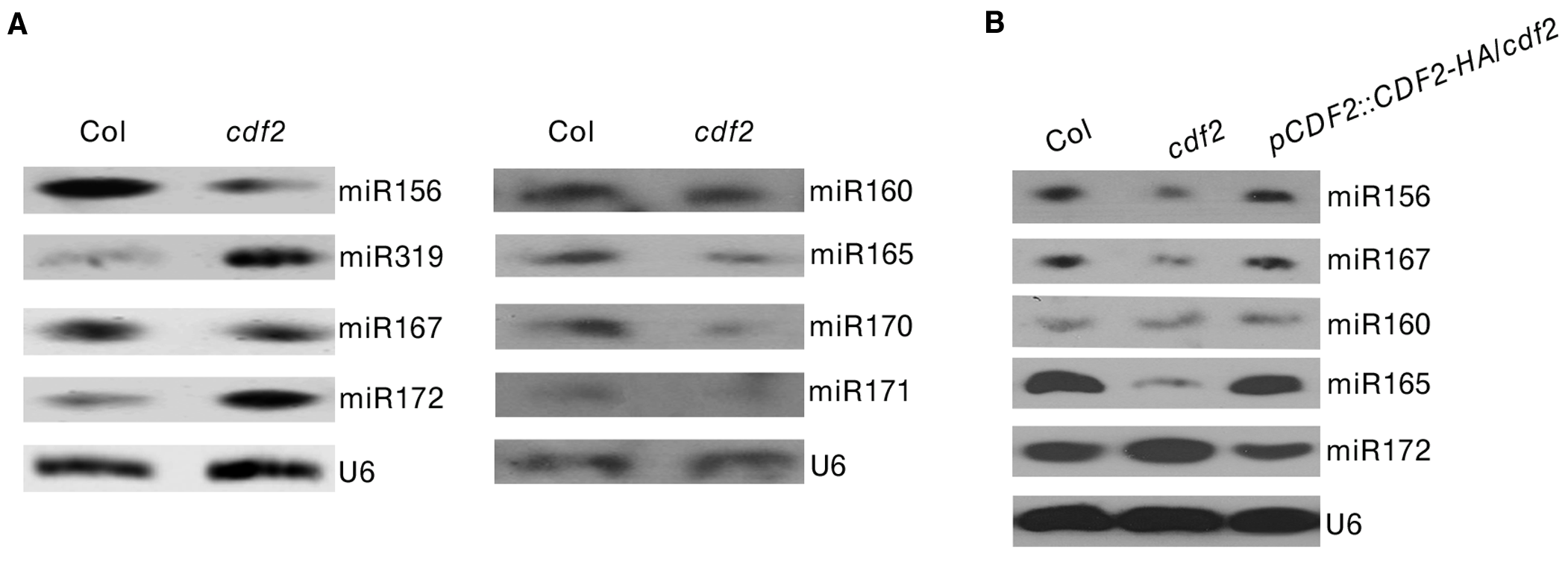

Supplement: S5 Fig — (A) Northern blots show the levels of miRNAs in 22-day-old seedlings of Col and cdf2 mutant. U6 serves as a loading control. (B) Northern blots show the levels of miRNAs in inflorescences of Col and cdf2 mutant. U6 serves as a loading control. (TIF) [file pgen.1005598.s005.tif]

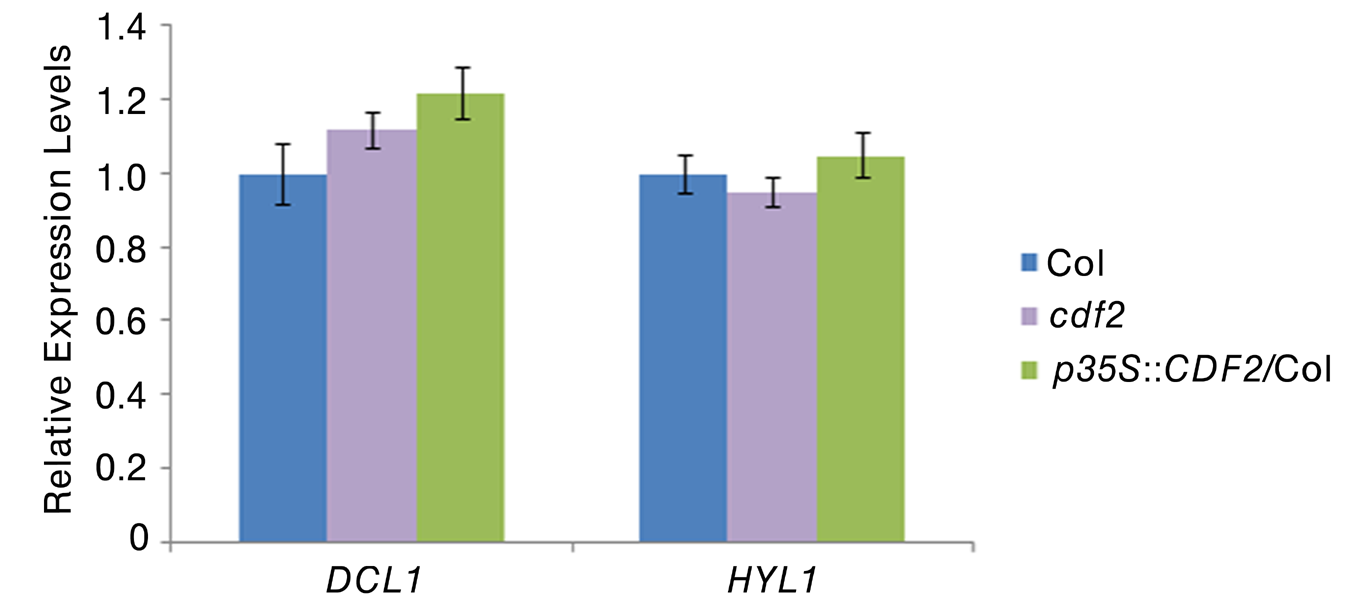

Supplement: S6 Fig — Data are given as means ± SD (n = 3). (TIF) [file pgen.1005598.s006.tif]

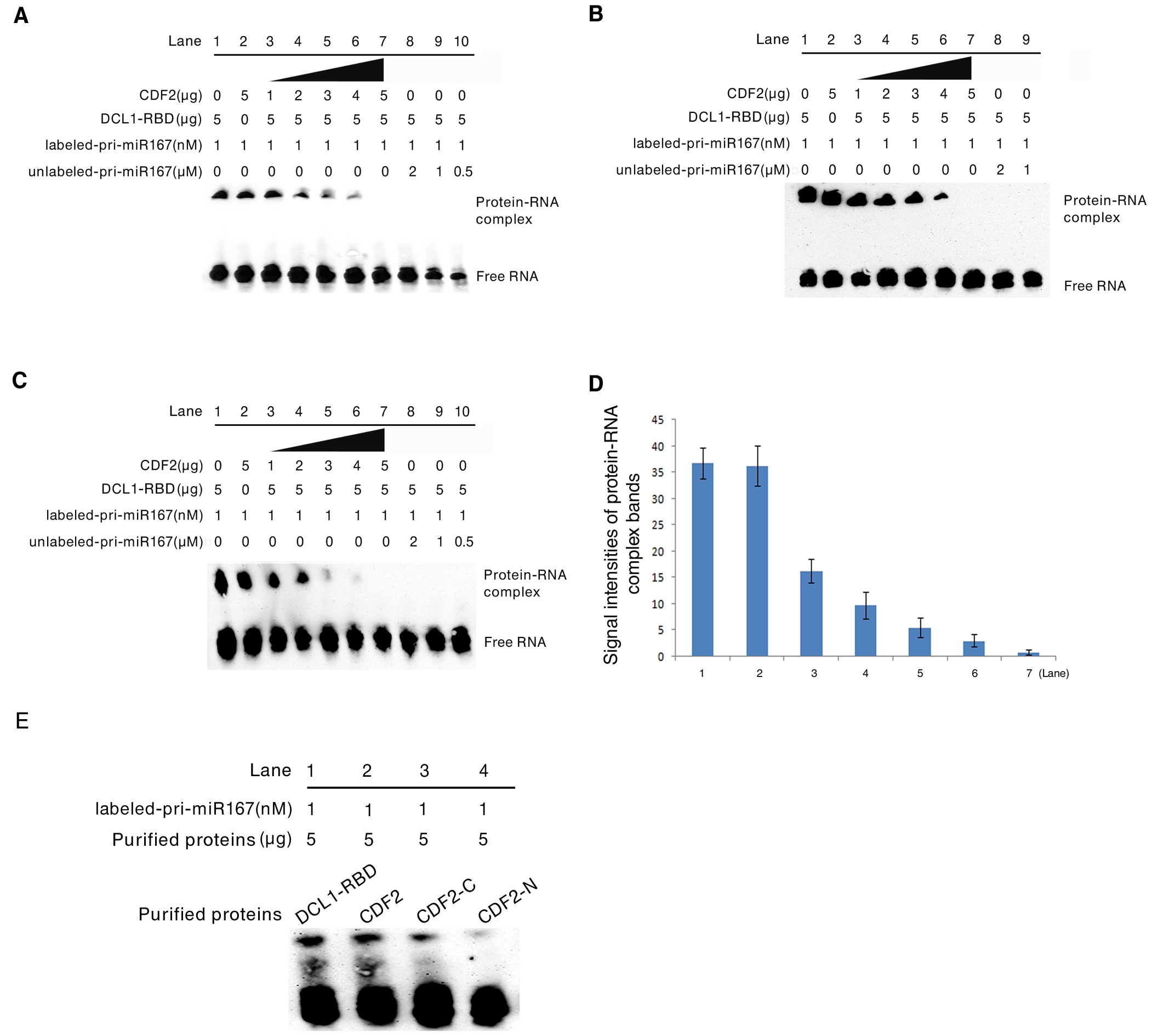

Supplement: S7 Fig — (A)-(C) RNA EMSAs show the effect of CDF2 on the binding of DCL1-RBD to pri-miRNA167. (D) The quantitative analysis of RNA binding activities of Lane1-7 in Figs 4A and S7A-S7C. (E) RNA EMSAs show the binding of CDF2 to pri-miRNA167 is mainly mediated by its C-terminal fragment. (TIF) [file pgen.1005598.s007.tif]

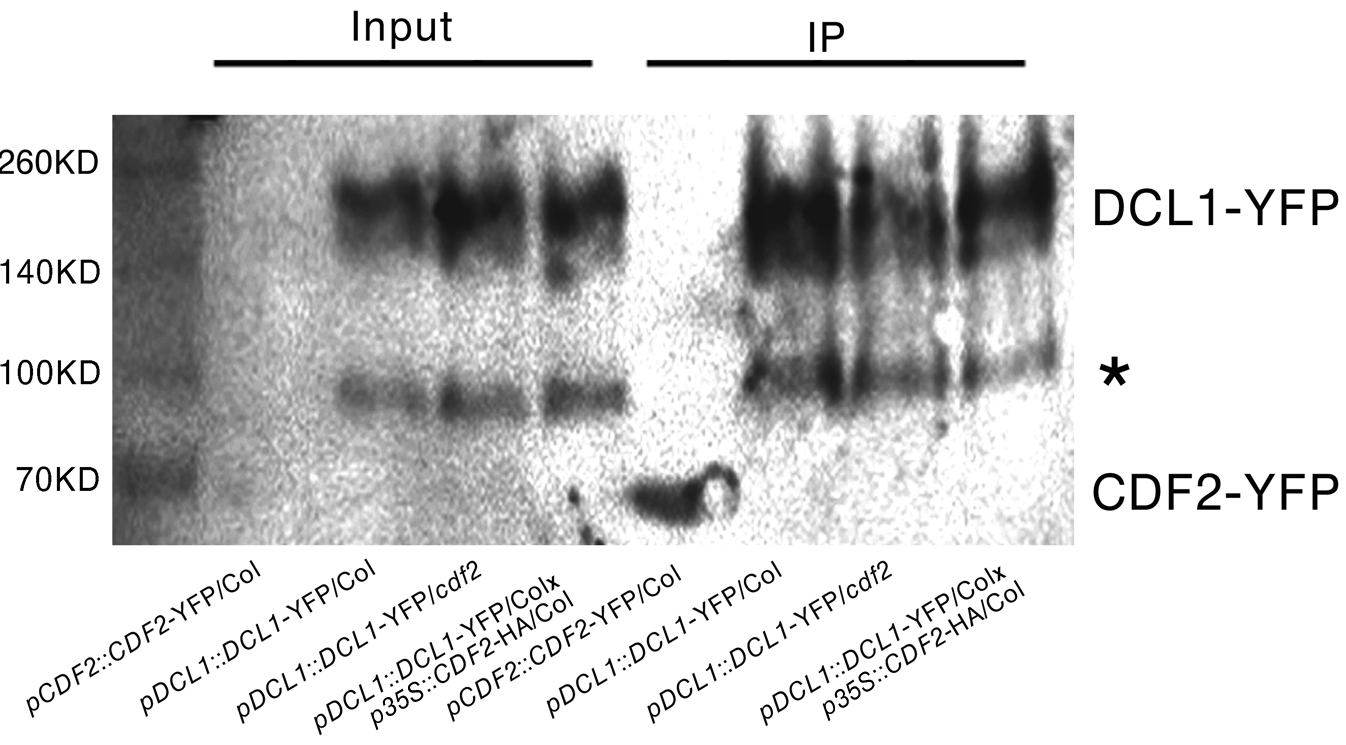

Supplement: S8 Fig — RIP assays were performed using 22-day-old plants, RNA fragments were immunoprecipitated with a GFP antibody. The CDF2-YFP and DCL1-YFP proteins were detected by an anti-GFP antibody.“*” stands for a nonspecific signal. (TIF) [file pgen.1005598.s008.tif]

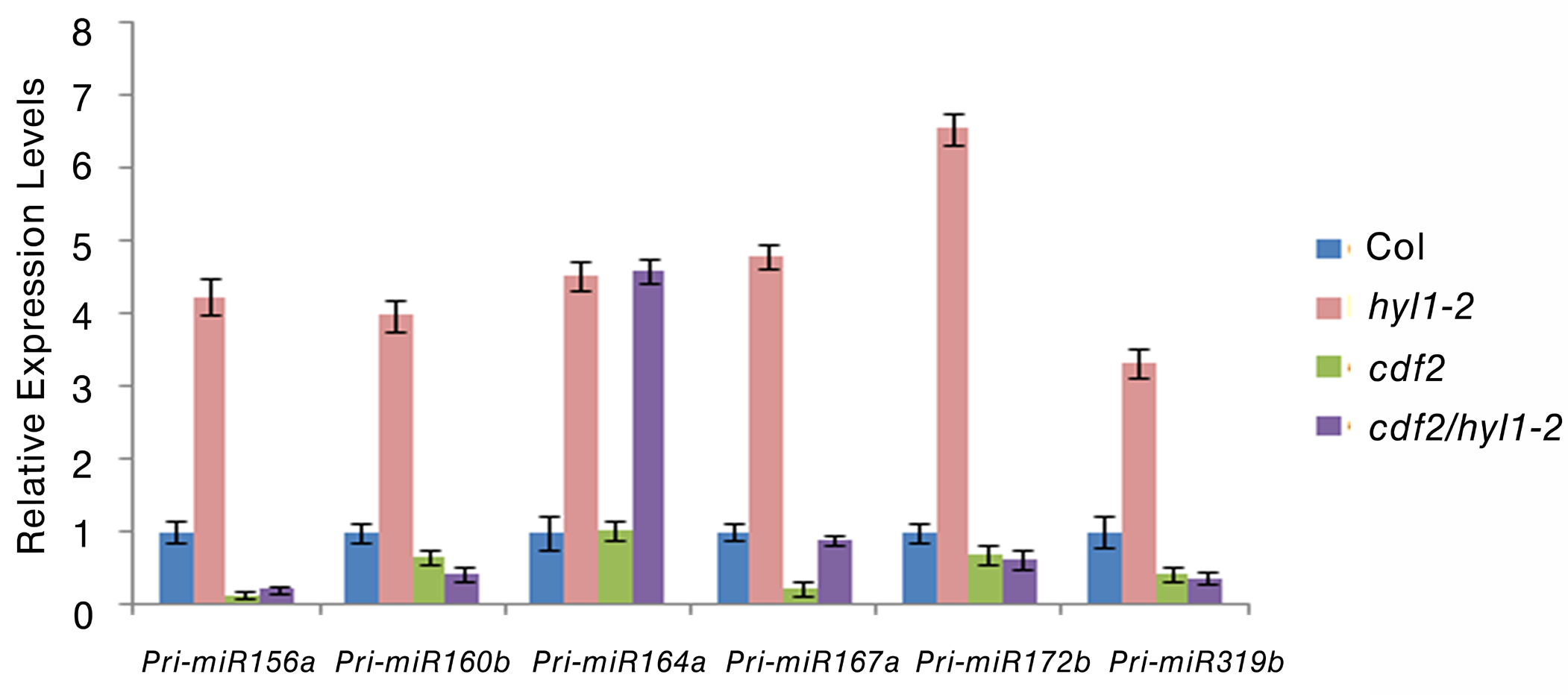

Supplement: S9 Fig — The relative fold changes were normalized to ACTIN. Data are given as means ± SD (n = 3). (TIF) [file pgen.1005598.s009.tif]

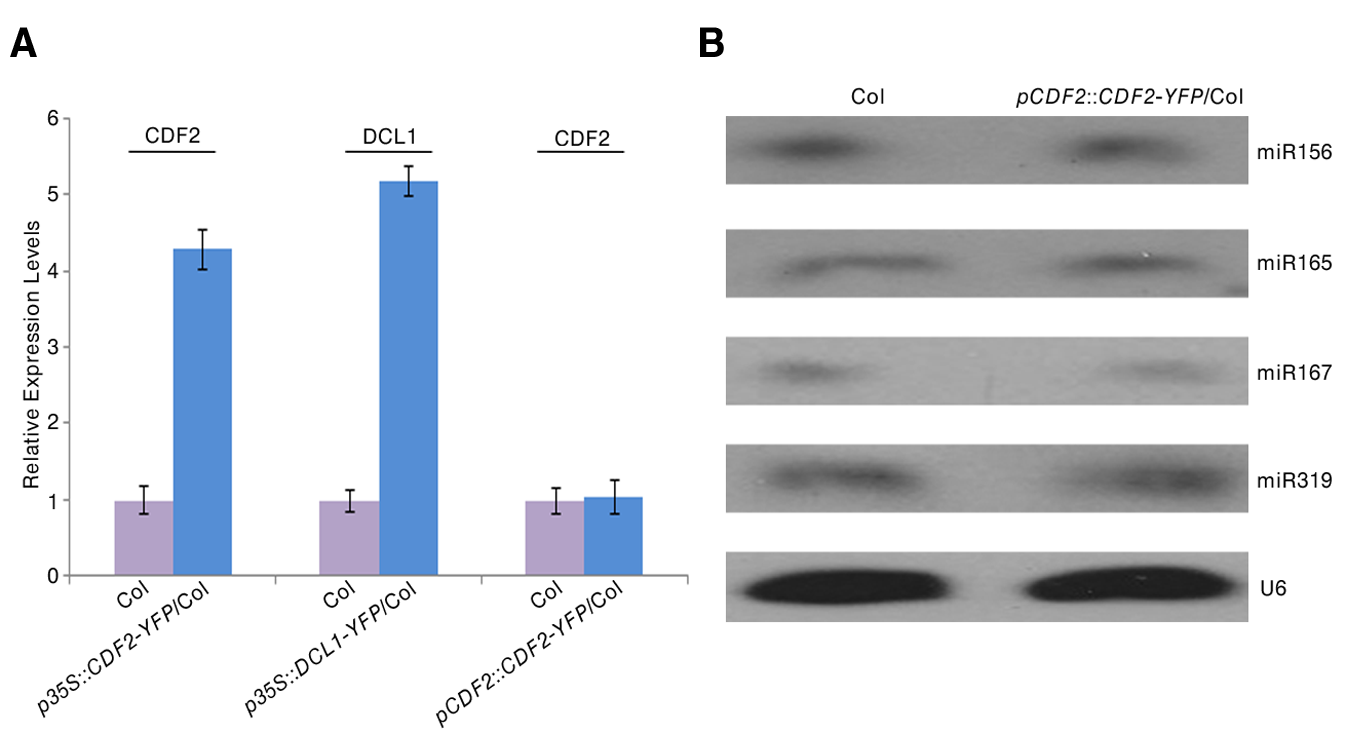

Supplement: S10 Fig — (A) The relative expression levels of CDF2, DCL1 and CDF2 in p35S::CDF2-YFP, p35S::DCL1-YFP and pCDF2::CDF2-YFP transgenic lines compared to corresponding genes in Col. Data are given as means ± SD (n = 3). (B) Northern blots show that the CDF2 transgenic lines driven by its endogenous promoter do not affect the miRNA accumulation. (TIF) [file pgen.1005598.s010.tif]

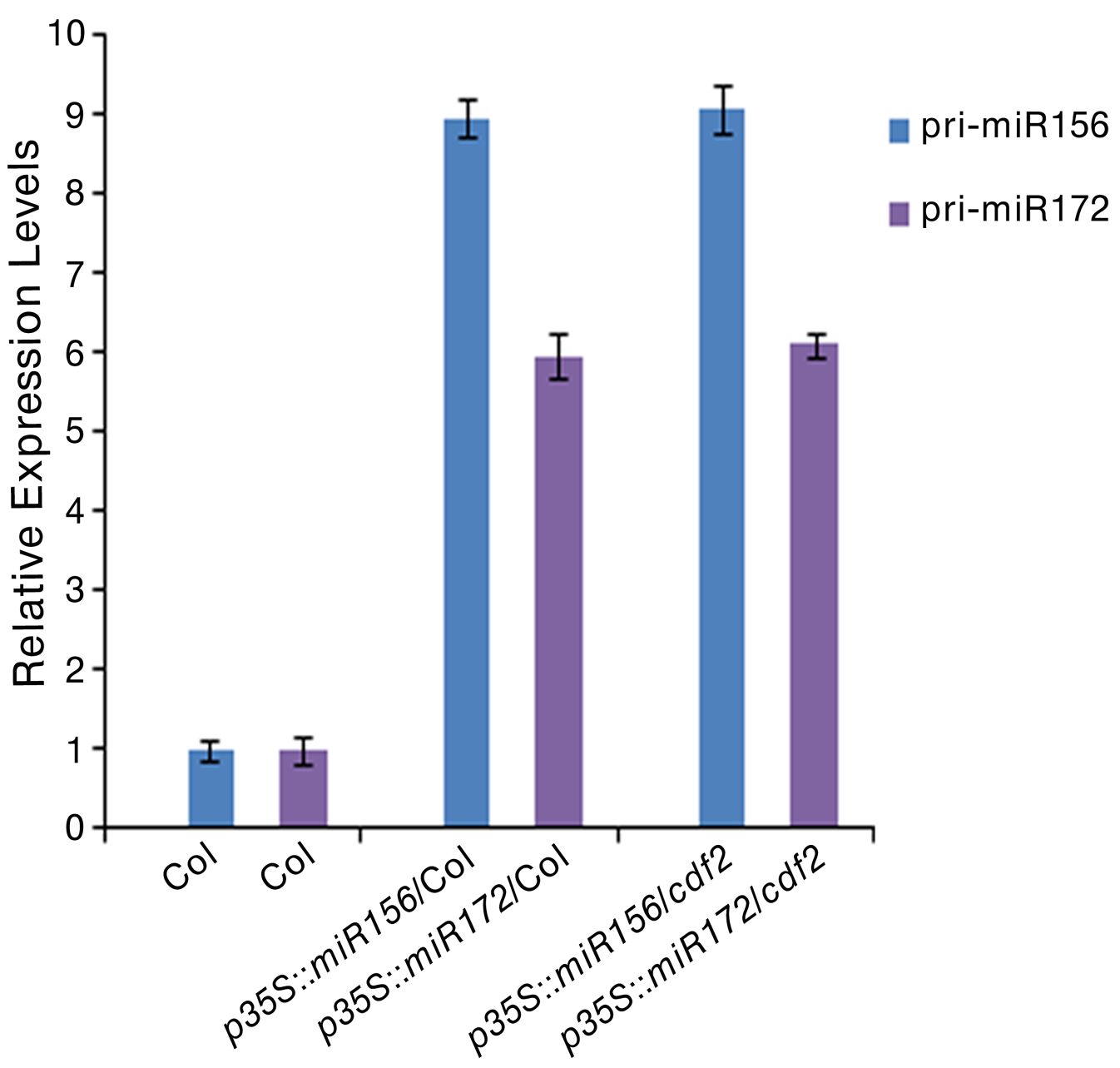

Supplement: S11 Fig — The relative expression levels of miR156 and miR172 in Col, p35S::miR156/Col and p35S::miR156/cdf2 or p35S::miR172/Col and p35S::miR172/cdf2 lines were detected by qRT-PCR. Data are given as means ± SD (n = 3). (TIF) [file pgen.1005598.s011.tif]

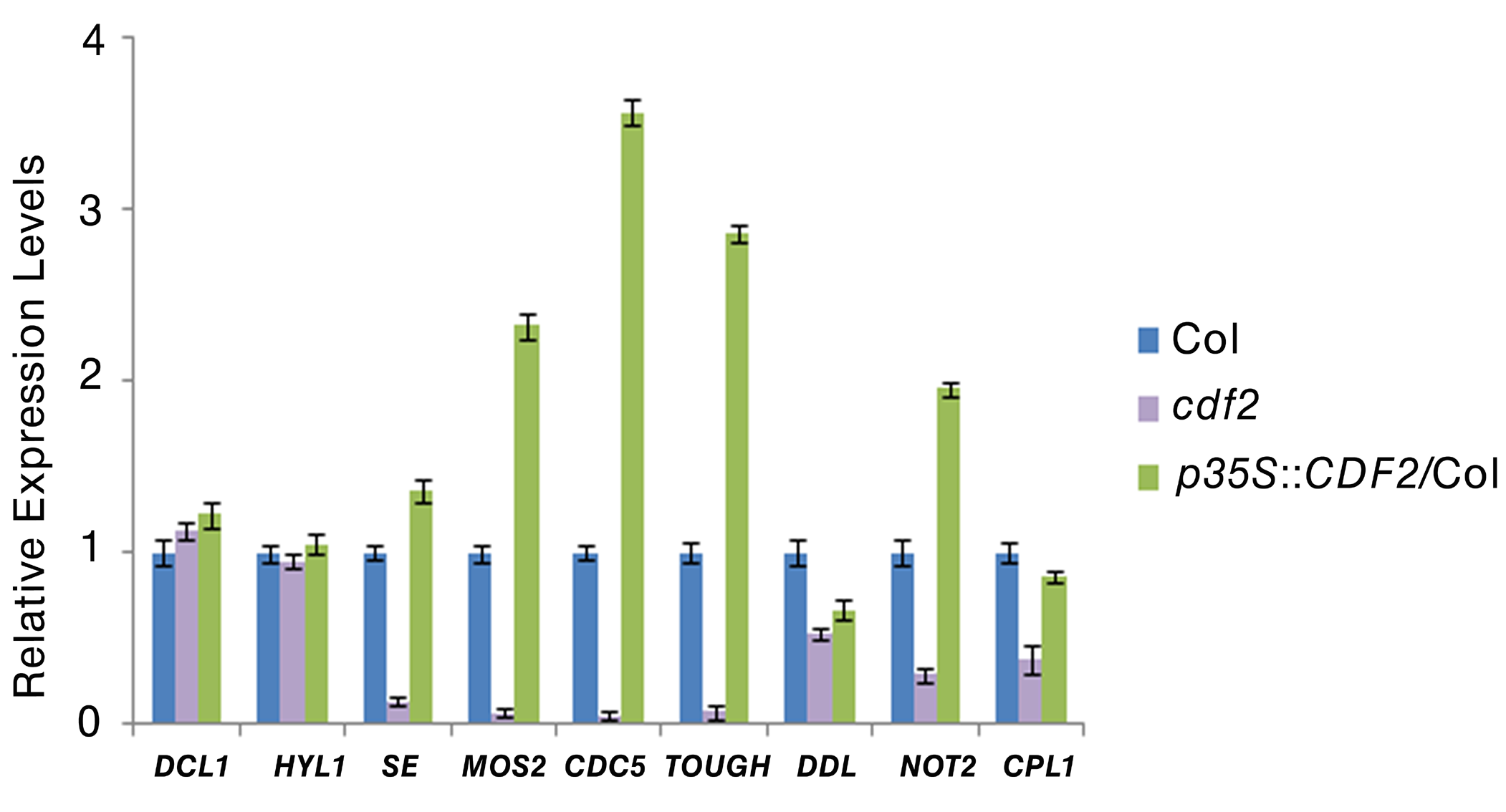

Supplement: S12 Fig — The relative expression levels of DCL1, HYL1, SE, MOS2, CDC5, TOUGH, DDL, NOT2, CPL1 were detected by qRT-PCR in Col wild type, cdf2 mutant and CDF2 overexpression lines. Data are given as means ± SD (n = 3). (TIF) [file pgen.1005598.s012.tif]

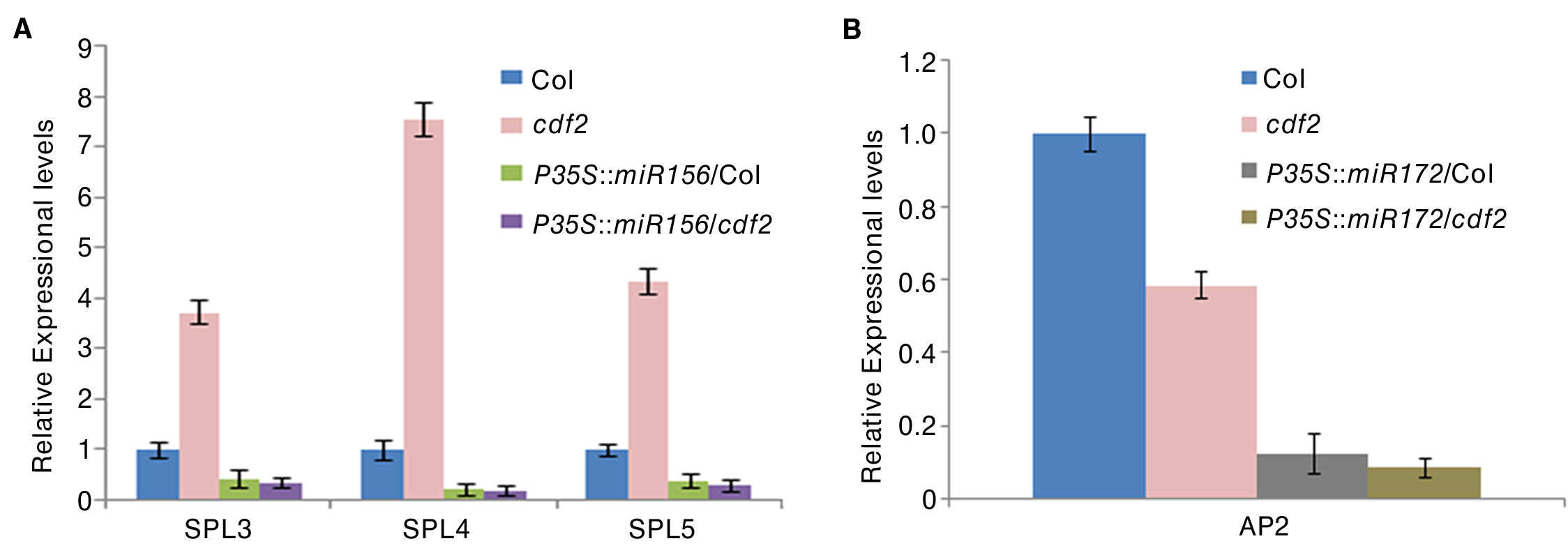

Supplement: S13 Fig — (A) The relative expressional levels of SPLs in Col, cdf2 mutant, p35S::miR156/Col and p35S::miR156/cdf2. (B) The relative expressional levels of AP2 in Col, cdf2 mutant, p35S::miR172/Col and p35S::miR172/cdf2. Data are given as means ± SD (n = 3). (TIF) [file pgen.1005598.s013.tif]

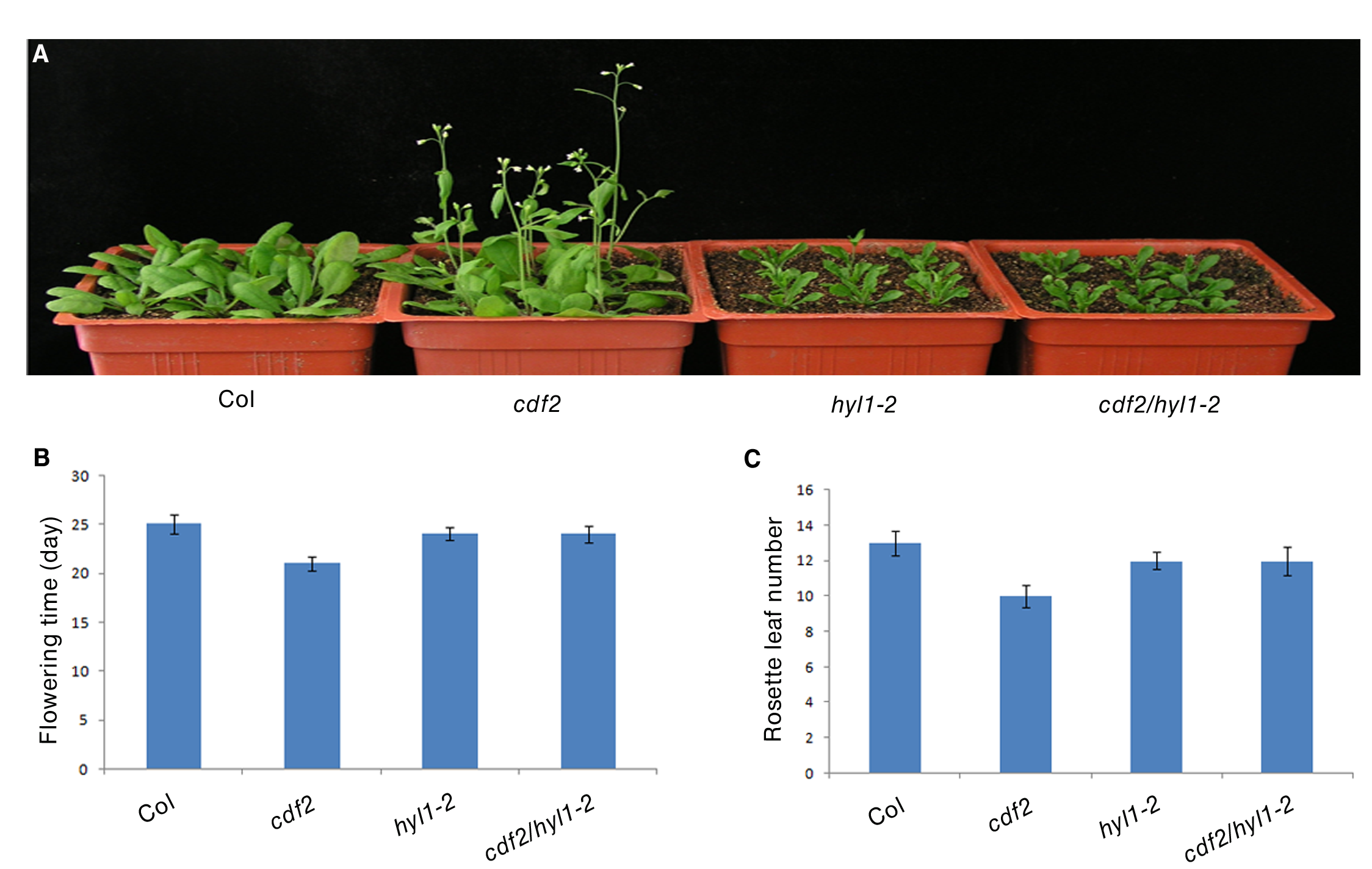

Supplement: S14 Fig — (A) 24-day-old plants of indicated genotypes grown in long day photoperiods (16 hours light, 8 hours dark). (B) The flowering time of the indicated genotypes shown in A. Data are mean ± SEM of 50 plants. (C) The number of rosette leaves at the time of flowering of the indicated genotypes shown in A. Data are means ± SEM of 50 plants. (TIF) [file pgen.1005598.s014.tif]
