# Supplementary material for: The Roles of Arabidopsis CDF2 in Transcriptional and Posttranscriptional Regulation of Primary MicroRNAs
Source: PLoS Genet. 2015 Oct 16;11(10):e1005598. doi: 10.1371/journal.pgen.1005598 (PMC4608766; doi:10.1371/journal.pgen.1005598)
Supplement: Supplementary file 2 [file pgen.1005598.s015.doc]

Table S1 The accumulation levels of miRNAs changed more than 1.5 fold in *cdf2* mutan

pairwise	miR-name	fold-change(log2 CDF/WT)	p-value		
WT-CDF	ath-miR156a	-0.8221788	0		
WT-CDF	ath-miR156b	-0.82196095	0		
WT-CDF	ath-miR156c	-0.8221788	0		
WT-CDF	ath-miR156d	-1.02580353	0		
WT-CDF	ath-miR156e	-0.82293135	0		
WT-CDF	ath-miR156f	-0.82291029	0		
WT-CDF	ath-miR156h	-0.77888173	0		
WT-CDF	ath-miR156j	-0.96769381	4.27E-32		
WT-CDF	ath-miR157a	-0.73615496	0		
WT-CDF	ath-miR157b	-0.73610417	0		
WT-CDF	ath-miR157c	-0.73554031	0		
WT-CDF	ath-miR160b	2.26590798	3.32E-09		
WT-CDF	ath-miR164c	-1.93431379	4.63E-170		
WT-CDF	ath-miR165a	-0.95915646	0		
WT-CDF	ath-miR165b	-0.95857842	0		
WT-CDF	ath-miR166a	-0.85209506	0		
WT-CDF	ath-miR166b	-0.8537039	0		
WT-CDF	ath-miR166c	-0.85378606	0		
WT-CDF	ath-miR166d	-0.85379205	0		
WT-CDF	ath-miR166e	-0.85365245	0		
WT-CDF	ath-miR166f	-0.85364946	0		
WT-CDF	ath-miR166g	-0.85373161	0		
WT-CDF	ath-miR167a	-0.82882493	0		
WT-CDF	ath-miR167b	-0.82913559	0		
WT-CDF	ath-miR167c	-0.75363042	0.01523158		
WT-CDF	ath-miR167d	-0.90491018	3.73E-129		
WT-CDF	ath-miR170	-1.53623205	0.01948937		
WT-CDF	ath-miR171a	-0.86995419	1.73E-12		
WT-CDF	ath-miR172b*	1.44237064	8.24E-233		
WT-CDF	ath-miR1888b	1.04431892	0.07067519		
WT-CDF	ath-miR2111a	-1.44871696	0.00189708		
WT-CDF	ath-miR2111b	-1.44871696	0.00189708		
WT-CDF	ath-miR2933a	0.84495917	0.00772682		
WT-CDF	ath-miR2933b	0.84495917	0.00772682		

WT-CDF	ath-miR2933b	0.84495917	0.00772682		
WT-CDF	ath-miR2937	-0.71168209	0.22367666		
WT-CDF	ath-miR319b	2.88194209	1.06E-14		
WT-CDF	ath-miR391	-1.46728324	0		
WT-CDF	ath-miR394a	4.05355152	2.73E-12		
WT-CDF	ath-miR394b	4.05355152	2.73E-12		
WT-CDF	ath-miR395b	0.86536628	0.00274767		
WT-CDF	ath-miR395c	0.86536628	0.00274767		
WT-CDF	ath-miR395f	0.86536628	0.00274767		
WT-CDF	ath-miR396a	-0.8151449	0		
WT-CDF	ath-miR396b	-0.81983855	0		
WT-CDF	ath-miR398a	-2.44906339	0.00217612		
WT-CDF	ath-miR398b	-1.959643	0.0033947		
WT-CDF	ath-miR398c	-1.959643	0.0033947		
WT-CDF	ath-miR399a	-2.23524957	0.00043425		
WT-CDF	ath-miR399d	-3.12154098	0.00033254		
WT-CDF	ath-miR399f	-1.7165304	1.06E-06		
WT-CDF	ath-miR4243	-1.18577264	0.03663906		
WT-CDF	ath-miR472	-1.35566878	0.0204595		
WT-CDF	ath-miR5020b	-1.22790486	9.24E-16		
WT-CDF	ath-miR5020c	-0.9081878	0.00479235		
WT-CDF	ath-miR5646	0.85936056	0.07043353		
WT-CDF	ath-miR5648-5p	-1.61863739	0.00062206		
WT-CDF	ath-miR5652	-1.0810913	0.01498338		
WT-CDF	ath-miR5657	0.70319614	0.13585813		
WT-CDF	ath-miR771	-1.48270735	1.30E-13		
WT-CDF	ath-miR783	-0.85683386	0.08515646		
WT-CDF	ath-miR822	-0.80850198	7.40E-59		
WT-CDF	ath-miR827	-1.12999134	0.0003343		
WT-CDF	ath-miR841b	-1.20364713	1.77E-06		
WT-CDF	ath-miR845a	-1.03376606	0.09552824		
WT-CDF	ath-miR851-5p	-0.70106965	0.1158062		
WT-CDF	ath-miR854a	0.92778491	5.40E-05		
WT-CDF	ath-miR854b	0.92778491	5.40E-05		
WT-CDF	ath-miR854c	0.92778491	5.40E-05		
WT-CDF	ath-miR854d	0.92778491	5.40E-05		
WT-CDF	ath-miR854e	0.92778491	5.40E-05		
WT-CDF	ath-miR857	1.23276718	4.68E-10		
WT-CDF	ath-miR869.2	1.064508	1.27E-59		
