# Supplementary material for: The Roles of Arabidopsis CDF2 in Transcriptional and Posttranscriptional Regulation of Primary MicroRNAs
Source: PLoS Genet. 2015 Oct 16;11(10):e1005598. doi: 10.1371/journal.pgen.1005598 (PMC4608766; doi:10.1371/journal.pgen.1005598)
Supplement: Supplementary file 3 [file pgen.1005598.s016.doc]

Table S2 The accumulation levels of miRNAs changed more than 1.5 fold in *dcl1-9* mutant

pairwise	miR-name	fold-chang	p-value
WT-DCL1-9	ath-miR156a	-4.26543	0
WT-DCL1-9	ath-miR156b	-4.26453	0
WT-DCL1-9	ath-miR156c	-4.26543	0
WT-DCL1-9	ath-miR156d	-5.28617	0
WT-DCL1-9	ath-miR156e	-4.27213	0
WT-DCL1-9	ath-miR156f	-4.27213	0
WT-DCL1-9	ath-miR156g	-5.63728	3.46E-89
WT-DCL1-9	ath-miR156h	-9.33654	0
WT-DCL1-9	ath-miR156j	-4.30461	3.00E-148
WT-DCL1-9	ath-miR157a	-5.76811	0
WT-DCL1-9	ath-miR157b	-5.76811	0
WT-DCL1-9	ath-miR157c	-5.77791	0
WT-DCL1-9	ath-miR157d	-7.14115	0
WT-DCL1-9	ath-miR158a	-9.058	0
WT-DCL1-9	ath-miR158b	-12.1144	8.58E-140
WT-DCL1-9	ath-miR159a	-6.64081	0
WT-DCL1-9	ath-miR159b	-8.05371	0
WT-DCL1-9	ath-miR159c	-9.19254	1.51E-192
WT-DCL1-9	ath-miR160a	-1.29894	9.61E-07
WT-DCL1-9	ath-miR160c	-1.35736	2.32E-07
WT-DCL1-9	ath-miR161.1	-3.94578	0
WT-DCL1-9	ath-miR161.2	-6.55583	0
WT-DCL1-9	ath-miR162a	-7.6186	0
WT-DCL1-9	ath-miR162b	-7.84032	0
WT-DCL1-9	ath-miR163	-5.72531	2.83E-95
WT-DCL1-9	ath-miR164a	-6.1952	0
WT-DCL1-9	ath-miR164b	-6.24915	0
WT-DCL1-9	ath-miR164c	-5.72391	0
WT-DCL1-9	ath-miR165a	-4.92671	0
WT-DCL1-9	ath-miR165b	-4.9074	0
WT-DCL1-9	ath-miR166a	-3.1838	0
WT-DCL1-9	ath-miR166b	-3.16249	0
WT-DCL1-9	ath-miR166c	-3.16262	0
WT-DCL1-9	ath-miR166d	-3.16262	0
WT-DCL1-9	ath-miR166e	-3.16225	0
WT-DCL1-9	ath-miR166f	-3.16221	0
WT-DCL1-9	ath-miR166g	-3.16237	0
WT-DCL1-9	ath-miR167a	-6.10205	0
WT-DCL1-9	ath-miR167b	-6.1026	0
WT-DCL1-9	ath-miR167c	-8.63633	3.90E-13
WT-DCL1-9	ath-miR167d	-5.30819	0
WT-DCL1-9	ath-miR168a	-2.33477	0
WT-DCL1-9	ath-miR168b	-2.33465	0
WT-DCL1-9	ath-miR169a	-5.5492	1.39E-124
WT-DCL1-9	ath-miR169b	-2.36316	7.76E-06
WT-DCL1-9	ath-miR169c	-7.41397	5.33E-06
[illegible]	[illegible]	[illegible]	[illegible]

WT-DCL1-9	ath-miR169e	-12.707	########
WT-DCL1-9	ath-miR169f	-12.6993	########
WT-DCL1-9	ath-miR169g-3	-11.6136	5.50E-99
WT-DCL1-9	ath-miR169g-5	-12.6963	########
WT-DCL1-9	ath-miR169h	-8.63633	3.90E-13
WT-DCL1-9	ath-miR169i	-10.3912	8.58E-43
WT-DCL1-9	ath-miR169j	-10.3912	8.58E-43
WT-DCL1-9	ath-miR169k	-8.63633	3.90E-13
WT-DCL1-9	ath-miR169l	-10.3912	8.58E-43
WT-DCL1-9	ath-miR169m	-8.71163	8.35E-14
WT-DCL1-9	ath-miR169n	-10.3912	8.58E-43
WT-DCL1-9	ath-miR170	-3.51185	0.001594
WT-DCL1-9	ath-miR171a	-4.77028	3.09E-68
WT-DCL1-9	ath-miR171b	-8.2213	5.16E-10
WT-DCL1-9	ath-miR171c	-8.2213	5.16E-10
WT-DCL1-9	ath-miR172a	-6.80773	0
WT-DCL1-9	ath-miR172b-3	-6.81535	0
WT-DCL1-9	ath-miR172b-5	-6.0403	########
WT-DCL1-9	ath-miR172c	-6.75974	########
WT-DCL1-9	ath-miR172d	-6.75751	########
WT-DCL1-9	ath-miR172e	-5.7819	1.15E-66
WT-DCL1-9	ath-miR173-3p	-6.79068	1.30E-35
WT-DCL1-9	ath-miR173-5p	-9.42434	0
WT-DCL1-9	ath-miR1886.1	-6.91648	0.000194
WT-DCL1-9	ath-miR1886.2	-10.6234	3.76E-50
WT-DCL1-9	ath-miR1888a	-9.71163	5.83E-27
WT-DCL1-9	ath-miR2111a-	-2.42433	0.00018
WT-DCL1-9	ath-miR2111b-	-2.42433	0.00018
WT-DCL1-9	ath-miR2933a	-2.37856	0.00027
WT-DCL1-9	ath-miR2933b	-2.37856	0.00027
WT-DCL1-9	ath-miR2937	-6.82896	0.000324
WT-DCL1-9	ath-miR319a	-6.69114	3.46E-33
WT-DCL1-9	ath-miR3440b-	-4.60757	9.35E-21
WT-DCL1-9	ath-miR390a	-3.61908	0
WT-DCL1-9	ath-miR390b	-3.62222	0
WT-DCL1-9	ath-miR391	-13.6463	0
WT-DCL1-9	ath-miR3932b	-4.2317	1.13E-10
WT-DCL1-9	ath-miR3933	-9.45835	1.01E-22
WT-DCL1-9	ath-miR395a	-7.47281	3.19E-06
WT-DCL1-9	ath-miR395b	-3.04883	5.21E-06
WT-DCL1-9	ath-miR395c	-3.04883	5.21E-06
WT-DCL1-9	ath-miR395d	-7.47281	3.19E-06
WT-DCL1-9	ath-miR395e	-7.47281	3.19E-06
WT-DCL1-9	ath-miR395f	-3.04883	5.21E-06
WT-DCL1-9	ath-miR396a	-10.1278	0
WT-DCL1-9	ath-miR396b	-10.0973	0
WT-DCL1-9	ath-miR397a	-6.1248	3.33E-22
WT-DCL1-9	ath-miR397b	-9.99891	9.28E-33
WT-DCL1-9	ath-miR398a	-6.82896	0.000324
WT-DCL1-9	ath-miR398b	-3.67229	0.000627
WT-DCL1-9	ath-miR398c	-3.67229	0.000627

WT-DCL1-9	ath-miR399a	-7.35253	8.90E-06					
WT-DCL1-9	ath-miR399b	-6.04494	########					
WT-DCL1-9	ath-miR399c	-8.36687	########					
WT-DCL1-9	ath-miR399d	-2.51185	0.006937					
WT-DCL1-9	ath-miR399f	-4.35509	1.02E-11					
WT-DCL1-9	ath-miR400	-3.74628	2.25E-07					
WT-DCL1-9	ath-miR402	-11.0674	5.44E-68					
WT-DCL1-9	ath-miR403	-8.808	0					
WT-DCL1-9	ath-miR408	-4.52998	6.28E-87					
WT-DCL1-9	ath-miR4243	-7.15086	4.15E-05					
WT-DCL1-9	ath-miR447a.2	-8.58387	1.09E-12					
WT-DCL1-9	ath-miR447a-3	-6.93214	2.32E-39					
WT-DCL1-9	ath-miR447b	-6.93214	2.32E-39					
WT-DCL1-9	ath-miR472	-7.15086	4.15E-05					
WT-DCL1-9	ath-miR5014a-	-7.26984	5.79E-50					
WT-DCL1-9	ath-miR5014b	-2.9604	1.26E-08					
WT-DCL1-9	ath-miR5020a	-4.17924	1.75E-19					
WT-DCL1-9	ath-miR5020b	-7.50117	8.16E-59					
WT-DCL1-9	ath-miR5020c	-4.20573	1.83E-10					
WT-DCL1-9	ath-miR5023	-8.2884	1.85E-10					
WT-DCL1-9	ath-miR5026	-7.0718	0					
WT-DCL1-9	ath-miR5629	-1.92688	0.02124					
WT-DCL1-9	ath-miR5635a	-2.05008	1.32E-19					
WT-DCL1-9	ath-miR5635b	-2.15662	3.18E-28					
WT-DCL1-9	ath-miR5635c	-2.15662	3.18E-28					
WT-DCL1-9	ath-miR5635d	-2.41916	5.46E-57					
WT-DCL1-9	ath-miR5641	-4.9479	1.31E-09					
WT-DCL1-9	ath-miR5642b	-1.02172	3.08E-12					
WT-DCL1-9	ath-miR5643a	-2.57975	3.74E-12					
WT-DCL1-9	ath-miR5643b	-2.42446	4.62E-12					
WT-DCL1-9	ath-miR5644	-2.42448	1.22E-37					
WT-DCL1-9	ath-miR5645a	-3.65078	########					
WT-DCL1-9	ath-miR5645b	-3.65078	########					
WT-DCL1-9	ath-miR5645c	-8.97874	1.76E-16					
WT-DCL1-9	ath-miR5645d	-3.87713	########					
WT-DCL1-9	ath-miR5645e	-4.16426	########					
WT-DCL1-9	ath-miR5645f	-3.68081	########					
WT-DCL1-9	ath-miR5647	-7.07692	6.94E-05					
WT-DCL1-9	ath-miR5648-3	-9.39883	7.84E-22					
WT-DCL1-9	ath-miR5648-5	-7.87338	5.24E-08					
WT-DCL1-9	ath-miR5652	-7.78319	1.46E-07					
WT-DCL1-9	ath-miR5653	-2.86778	5.08E-67					
WT-DCL1-9	ath-miR5654-3	-4.2317	8.64E-06					
WT-DCL1-9	ath-miR5655	-2.91621	7.54E-07					
WT-DCL1-9	ath-miR5656	-4.17923	7.01E-15					
WT-DCL1-9	ath-miR5657	-2.3313	0.015883					
WT-DCL1-9	ath-miR5659	-2.04993	1.01E-22					
WT-DCL1-9	ath-miR5661	-8.52943	3.04E-12					
WT-DCL1-9	ath-miR5663	-9.73588	2.09E-27					
WT-DCL1-9	ath-miR5666	-6.82896	0.000324					

WT-DCL1-9	ath-miR5995b	-3.59424	0.001001					
WT-DCL1-9	ath-miR5996	-6.35532	########					
WT-DCL1-9	ath-miR5997	-8.35253	6.62E-11					
WT-DCL1-9	ath-miR5999	-7.07692	6.94E-05					
WT-DCL1-9	ath-miR771	-10.2553	5.30E-39					
WT-DCL1-9	ath-miR773a	-2.88379	3.00E-05					
WT-DCL1-9	ath-miR775	-12.9151	########					
WT-DCL1-9	ath-miR777	-7.78319	1.46E-07					
WT-DCL1-9	ath-miR779.2	-7.07692	6.94E-05					
WT-DCL1-9	ath-miR780.1	-8.00781	0					
WT-DCL1-9	ath-miR780.2	-7.82753	0					
WT-DCL1-9	ath-miR783	-3.9479	9.47E-05					
WT-DCL1-9	ath-miR822	-2.74509	########					
WT-DCL1-9	ath-miR823	-4.09677	3.06E-18					
WT-DCL1-9	ath-miR824	-3.01345	4.26E-48					
WT-DCL1-9	ath-miR825	-5.19914	4.78E-43					
WT-DCL1-9	ath-miR827	-8.78319	1.79E-14					
WT-DCL1-9	ath-miR829.1	-6.85062	3.73E-37					
WT-DCL1-9	ath-miR832-5r	-8.47285	8.48E-12					
WT-DCL1-9	ath-miR833a-5	-10.0958	5.47E-35					
WT-DCL1-9	ath-miR833b	-7.28845	1.49E-05					
WT-DCL1-9	ath-miR837-3r	-9.4289	2.81E-22					
WT-DCL1-9	ath-miR841a	-9.16882	1.04E-18					
WT-DCL1-9	ath-miR841b-3	-5.69114	2.60E-62					
WT-DCL1-9	ath-miR841b-5	-6.05371	4.14E-21					
WT-DCL1-9	ath-miR843	-9.4872	3.60E-23					
WT-DCL1-9	ath-miR845a	-6.82896	0.000324					
WT-DCL1-9	ath-miR846	-5.19253	6.10E-22					
WT-DCL1-9	ath-miR848	-7.67465	########					
WT-DCL1-9	ath-miR850	-10.9989	7.20E-65					
WT-DCL1-9	ath-miR851-3r	-11.2678	5.02E-78					
WT-DCL1-9	ath-miR851-5r	-7.58383	1.14E-06					
WT-DCL1-9	ath-miR852	-10.0958	5.47E-35					
WT-DCL1-9	ath-miR854a	-3.25725	3.75E-09					
WT-DCL1-9	ath-miR854b	-3.25725	3.75E-09					
WT-DCL1-9	ath-miR854c	-3.25725	3.75E-09					
WT-DCL1-9	ath-miR854d	-3.25725	3.75E-09					
WT-DCL1-9	ath-miR854e	-3.25725	3.75E-09					
WT-DCL1-9	ath-miR857	-8.93752	4.92E-16					
WT-DCL1-9	ath-miR860	-8.97874	1.76E-16					
WT-DCL1-9	ath-miR861-5r	-6.73593	0.000541					
WT-DCL1-9	ath-miR863-3r	-6.88992	########					
WT-DCL1-9	ath-miR864-5r	-2.92682	1.94E-05					
WT-DCL1-9	ath-miR866-5r	-8.2213	5.16E-10					
WT-DCL1-9	ath-miR869.2	-8.80589	########					
