# Supplementary material for: The Roles of Arabidopsis CDF2 in Transcriptional and Posttranscriptional Regulation of Primary MicroRNAs
Source: PLoS Genet. 2015 Oct 16;11(10):e1005598. doi: 10.1371/journal.pgen.1005598 (PMC4608766; doi:10.1371/journal.pgen.1005598)
Supplement: Supplementary file 4 [file pgen.1005598.s017.doc]

PGREEN-62-SK-CDF2-F	AAACTAGTATGGCTGATCCGGCGATTAA	A dual luciferase reporter assay	sense
PGREEN-62-SK-CDF2-R	AAAAGCTTCTATGAGCTCTCATGGAAGT	A dual luciferase reporter assay	antisense
PGREEN-62-SK-DCL1-RBD-F	AAACTAGTatgcatccggtgcgagagct	A dual luciferase reporter assay	sense
PGREEN-62-SK-DCL1-RBD-R	AAAAGCTTAGAAAAAGTTTTATTTAAAAGC	A dual luciferase reporter assay	antisense
PGREEN-0800-LUC-PRO172-F	AATCTAGAtaatctactattaatatta	A dual luciferase reporter assay	sense
PGREEN-0800-LUC-PRO172-R	AAGCTTCTACAAACAACGACAGATGAGCT	A dual luciferase reporter assay	antisense
PGREEN-0800-LUC-PRO156-F	AATCTAGAccataaatatggttcggtcc	A dual luciferase reporter assay	sense
PGREEN-0800-LUC-PRO156-R	AAAAGCTTCTACAAACAACGACAGATGA	A dual luciferase reporter assay	antisense
Actine-F	GGTAACATTGTGCTCAGTGGTGG	Real time PCR	sense
Actine-R	AACGACCTTAATCTTCATGCTGC	Real time PCR	antisense
HYL1-F	ATGACCTCCACTGATGTTTCCTC	Real time PCR	sense
HYL1-R	CATACTCCTGCAACCGAC	Real time PCR	antisense
DCL1-F	ATGCATCCGGTGCGAGAGCTA	Real time PCR	sense
DCL1-R	TTATATTTCTTTCTCTTTCAAAG	Real time PCR	antisense
pri-mi156d-F	GAGATAGACAATAAATTCGATGAGA	Real time PCR	sense
pri-mi156d-R	TTTTCATCGGTTTCTGG	Real time PCR	antisense
pri-mi156e-F	CAAAGATAGAAAGATGTAAGGTCTA	Real time PCR	sense
pri-mi157e-R	TGTACTACTCATCCCTTCG	Real time PCR	antisense
pri-mi157a-F	ACCCTTCAATAGATATATGA	Real time PCR	sense
pri-mi157a-R	ATTCTCTTATATAAGCCTTA	Real time PCR	antisense
pri-mi157c-F	ACCGGCAATGATGAAGAATGTATAA	Real time PCR	sense
pri-mi157c-R	TTCCCTTTGTCACTTCATA	Real time PCR	antisense
pri-mi159a-F	ATTCTCAAATTATAGCGAAT	Real time PCR	sense
pri-mi159a-R	TAGTGTAAGAGATTTACCTC	Real time PCR	antisense
pri-mi160a-F	GGCTTCAAGAACAGTAACCC	Real time PCR	sense
pri-mi160a-R	AAAAAAGCATATGATGGATT	Real time PCR	antisense
pri-mi160c-F	TGATTCTTCTATAAAATATCAAATA	Real time PCR	sense
pri-mi160c-R	ACTACTTCATTTCCTCTTCC	Real time PCR	antisense
pri-mi165a-F	GTTTAAATACAAATAAACGAAACAA	Real time PCR	sense
pri-mi165a-R	GCCCATCTTCGTCTCC	Real time PCR	antisense
pri-mi166a-F	GACTCTGGCTCGCTCTATTCA	Real time PCR	sense
pri-mi166a-R	TGGTCCGAAGACGCTAAAAC	Real time PCR	antisense
pri-mi166f-F	AATTAGTGAGAGAGGAGAAAACAAA	Real time PCR	sense
pri-mi166f-R	TTGCTGCGGATTGATG	Real time PCR	antisense
pri-mi167a-F	GTTCTTTTTTTCTTTCTCTCGA	Real time PCR	sense
pri-mi167a-R	GCCTCAGAAAGAGAAGTAAGCT	Real time PCR	antisense
pri-mi167b-F	TTCTCTCATCTCCTTTCTGTA	Real time PCR	sense
pri-mi167b-R	TGAGAGACACCATTTTGGTATT	Real time PCR	antisense
pri-mi170-F	TCAATTAGAAAGGAGAAAGGAGCAT	Real time PCR	sense
pri-mi170-R	CAGAGTTATTAAATGCTTCG	Real time PCR	antisense
pri-mi171a-F	TAAAGAGGGTGAGGCCAGCTTT	Real time PCR	sense
pri-mi171a-R	TGGAAAGGAAGAAATGGAAA	Real time PCR	antisense
pri-mi172b-F	TCTCTTGTGCGTGCGTAAAT	Real time PCR	sense
pri-mi172b-R	CGCCTACAAACAACGACAGA	Real time PCR	antisense

pri-mi319b-F	TGCAAAACAACACTATAAACA	Real time PCR	sense
pri-mi319b-R	ATTGCATCCAGAATCACAAA	Real time PCR	antisense
pri-mi394a-F	AGCTTATATAGCCCGTCATA	Real time PCR	sense
pri-mi394a-R	CGAGAGAATCAAAGATGTGAG	Real time PCR	antisense
pri-mi394b-F	TCTCTCTCTCTCTATCTTTT	Real time PCR	sense
pri-mi394b-R	AGATCAAAGTTTGACAGAT	Real time PCR	antisense
pri-mi395b-F	CCAACATTTCGACTCGATTCATCTT	Real time PCR	sense
pri-mi395b-R	AACGGCTGCAATGGT	Real time PCR	antisense
pri-mi398b-F	TTCAGAAAAGTACAATGATG	Real time PCR	sense
pri-mi398b-R	ACAATAAGTAGATCCTTCACCAT	Real time PCR	antisense
GUS-F	CGATGTCACTCCGTATGTTATTG	Real time PCR	sense
GUS-R	CAGTTCTTTCGGCTTGTTGC	Real time PCR	antisense
SE-F	CTGACCTTGTCGATAACCGTCTCC	Real time PCR	sense
SE-R	CAGGCCTCCCACCCATTTCAC	Real time PCR	antisense
MOS2-F	ATTCGATCCA TCGAAAACCC	Real time PCR	sense
MOS2-R	TCCACAGGAACACTCTCAAA	Real time PCR	antisense
CDC5-F	TTCAGAGACC TTGAATTGGA	Real time PCR	sense
CDC5-R	AGCTTCTCATCTTCTTCTCT	Real time PCR	antisense
DDL-F	GAGAAGGAAA TTGGGAGAAG	Real time PCR	sense
DDL-R	ACCCCTCATCATGCCTTGAG	Real time PCR	antisense
TOUGH-F	AGAAATCGCA AGCCGGAAGA	Real time PCR	sense
TPUGH-R	TAAACCCAAATGTGTCAAAT	Real time PCR	antisense
NOT2-F	TTCCATCTGC TGGTGTCCAA	Real time PCR	sense
NOT2-R	CCATTGTGCCTCCACCAACC	Real time PCR	antisense
CPL-F	TGAATCAGCAACAAGATGAT	Real time PCR	sense
CPL-R	ATATTTTCCGAGTACATAGC	Real time PCR	antisense
pri-miR167 clone-F	ATTTCTCCACTTCTTGAGCTTCC	EMSA	sense
pri-miR167 clone-R	AGTCAACTGTGTGCGTTCGGGACT	EMSA	antisense
HYL-MBP-F	ATGACCTCCACTGATGTTTC	EMSA	sense
HYL1-MBP-R	GTCTGACTGGATCGCTAAAG	EMSA	antisense
CDF2-N-F	ATGGCTGATCCGGCGATTAAGCTC	BiFC and EMSA	sense
CDF2-N-R	GACTGTTCCAGCTTCTTCGTTTG	BiFC and EMSA	antisense
CDF2-C-F	AGCCCGTTACCAAAAGTTCCATGCTT	BiFC and EMSA	sense
CDF2-C-R	CTATGAGCTCTCATGGAAGTTTG	BiFC and EMSA	antisense
